# Association Between Means Restriction of Poison and Method-Specific Suicide Rates: A Systematic Review

**DOI:** 10.1001/jamahealthforum.2021.3042

**Published:** 2021-10-15

**Authors:** Jessy S. Lim, Nicholas A. Buckley, Kate M. Chitty, Rebekah Jane Moles, Rose Cairns

**Affiliations:** 1 Sydney Pharmacy School, Faculty of Medicine and Health, The University of Sydney, Sydney, New South Wales, Australia; 2 Discipline of Biomedical Informatics and Digital Health, Faculty of Medicine and Health, The University of Sydney, Sydney, New South Wales, Australia; 3 New South Wales Poisons Information Centre, The Children’s Hospital at Westmead, Sydney, New South Wales, Australia; 4 Edith Collins Centre (Translational Research in Alcohol Drugs and Toxicology), Sydney Local Health District, New South Wales, Australia

## Abstract

**Importance:**

Suicide is a major and preventable cause of death worldwide. Means restriction aims to reduce the rate of completed suicides by limiting public access to lethal suicide methods. Means restriction of agents used in poisoning is more controversial because there is potential to switch to other lethal methods.

**Objective:**

To identify the changes in suicide rate by the targeted poison and the corresponding changes in suicide by other methods.

**Evidence Review:**

Five databases (MEDLINE, Embase, Scopus, PsycInfo, and Web of Science) were searched for studies on national means restriction of poison that were published in the English language from inception until December 31, 2019. Of the 7657 articles that were screened by title and abstract, 62 studies were included in the analysis.

**Findings:**

Sixty-two studies from 26 countries in Europe, the US, Australia, Asia, and the United Kingdom were included in the review. The studies included restrictions on pesticides (15 countries), domestic gas (14 countries), motor vehicle exhaust (9 countries), and pharmaceuticals (8 countries). The median (IQR) change in method-specific suicide rates was −1.18 (−2.03 to −0.46) per 100 000 people after restrictions, whereas the median (IQR) change in other methods of suicide (not the restricted poison) was −0.09 (−2.22 to 1.65) per 100 000 people. Decreases in suicide by the restricted poison were not associated with increases in suicide by other methods. An estimated 57 355 poison-specific suicides annually (before the interventions were implemented) may have benefited the most from means restriction.

**Conclusions and Relevance:**

This systematic review found that means restriction of poison was associated with decreased method-specific suicide rates without an equivalent shift toward the use of other methods. Suicide prevention strategies need to address both individuals who are at risk and population-level policies.

## Key Points

**Question:**

What is the association between means restriction of poison and population-level suicide rates?

**Findings:**

In this systematic review of 62 studies from 26 countries, means restriction of poison was associated with reductions in method-specific suicide rates without an equivalent shift toward other methods. Means restriction was most promising when the poison was lethal and common, and decreases in suicides by the restricted poison were not associated with increases in suicide by other available methods.

**Meaning:**

The findings suggest that restricting access to poisons was associated with decreases in suicide by poisoning. Changes in other methods of suicide were associated with historical trends rather than reduced availability of the poison.

## Teaser

This systematic review reports the preintervention and postintervention changes in suicide rates using restricted methods, such as pesticides, domestic gas, motor vehicle exhaust, and pharmaceuticals, among individuals in 26 countries.

## Introduction

Suicide prevention is an urgent health priority worldwide, and 77% of suicides occur in low- and middle-income countries.^1^ Suicide is a leading cause of years of life lost in many countries, especially in adolescents and young adults.^2^ In addition, suicide has consequences for the person’s community, such as bereavement, stigma, and contagion.^3,4^

Although medical, psychosocial, and pharmacological treatments contribute to suicide prevention on an individual level, they are not accessed by everybody who is at risk for suicide. Population-level suicide prevention strategies protect people in a crisis, regardless of whether they seek medical or psychological help for their suicidal thoughts or behavior^3^; examples include media guidelines and means restriction.^5^

Restricting access to lethal and common means (means restriction) can lower the likelihood of a suicide attempt, delay a suicide attempt, or lead to use of a less lethal method of attempting suicide.^6^ Any of these outcomes are associated with a better chance of survival and rehabilitation given that many suicide attempts are impulsive, and people are often discouraged if their preferred method is not readily available.^6^

Means restrictions, such as bridge barriers and gun control laws, are associated with a reduced number of suicides by jumping and by firearms, respectively.^7,8,9^ Pesticides are responsible for up to 20% of global suicides, especially in low- and middle-income countries.^10,11^ Pesticide restrictions are also associated with decreased suicide rates, especially when the targeted pesticides were hazardous or frequently used.^10^ Suicide attempts by other poisons and medicines are common, but these means have not been evaluated as extensively.^11^

Although means restriction is usually associated with reduced incidence of method-specific suicide rates, a subsequent shift toward other methods of suicide is a possible outcome. This shift is known as method substitution or displacement and is often debated by epidemiologists and suicide prevention experts when considering the overall benefit of means restriction.^3,12,13^ Population-level suicide is inherently dynamic and affected by multiple factors, and changes in other methods of suicide may be coincidental or driven by changes in means restriction.

In this study, we aimed to assess population-level means restriction policies for poisons. Specifically, the goal was to identify changes in suicide rate by the targeted poison and, if available, the corresponding changes in suicide by other methods. We tested whether an increased incidence of other methods (method substitution) was consistently observed in these studies and whether it was associated with the change in suicide by the targeted poison.

## Methods

This study is registered with PROSPERO (CRD42020160734). Articles were extracted and screened for relevance according to the Preferred Reporting Items for Systematic Reviews and Meta-analyses (PRISMA) reporting guideline.^14^

### Search Strategy and Inclusion Criteria

We conducted a systematic review of international evidence to assess the association between means restriction and suicide by poisoning. We searched key words and/or Medical Subject Headings in 5 databases: MEDLINE, Embase, PsycInfo, Scopus, and Web of Science. The search strategy combined 3 concepts: suicide, poison, and restriction (eMethods 1 in the Supplement). Poison included terms such as overdose, medicine, and domestic chemicals. Restriction included terms such as intervention, policy, access, and scheduling. We retrieved all studies that were published in the English language from inception until December 31, 2019.

We excluded studies that were retrieved from gray literature, which we defined as literature searches that were not replicable (eg, Google Scholar) and works that were not published in a peer-reviewed, academic journal (eg, government reports). Literature reviews were also excluded; however, we read relevant reviews to check for additional studies. We searched for additional studies by citation chaining (assessing the references of relevant papers) and checking publications by prominent authors in the field of means restriction.

We selected longitudinal studies that measured changes in suicide rate while means restriction was implemented. The inclusion criteria were national interventions at the country level, and the outcomes were suicide rates reported in the country or a smaller, representative region.

Studies that discussed means restriction but did not report changes in suicide rates or that reported trends in suicide rates without a relevant intervention were excluded. We also excluded interventions that were conducted at a community or regional level, such as interventions limited to 1 province or city.

### Study Selection, Data Extraction, and Quality Assessment

Studies were exported into EndNote (Clarivate) and deduplicated. Unique articles were exported into Covidence (Veritas Health Innovation). Two of us (J.S.L. and R.C.) independently performed title and abstract screening. An article was reviewed for relevance in full text if a decision could not be readily made from the title and abstract alone. Conflicts were discussed with a third author (N.A.B.), who helped us reach consensus.

Two of us (J.S.L. and R.C.) also independently extracted data. Data extracted included study location, study design, years reported, years of intervention, details of intervention, and suicide rates by the poison of interest. We extracted data, when available, on overall suicide rates (suicide by any method) and suicide by other methods (not by the targeted poison). The double data extraction was checked for consistency, and a meeting was held to resolve any conflicts.

The main findings from each article are presented in a narrative synthesis (Table).^15,16,17,18,19,20,21,22,23,24,25,26,27,28,29,30,31,32,33,34,35,36,37,38,39,40,41,42,43,44,45,46,47,48,49,50,51,52,53,54,55,56,57,58,59,60,61,62,63,64,65,66,67,68,69,70,71,72,73,74,75,76^ We assessed each study for quality and bias using the Risk Of Bias In Nonrandomized Studies of Interventions (ROBINS-I) tool for uncontrolled before and after studies (eTable 1 in the Supplement).^77^

**Table.** Study Location, Intervention, and Changes in Method-Specific Suicide Rates, Overall Suicide Rates, and Other Suicide Methods^a^

Location	Year and intervention details	Change in suicide by poison of interest (reported until the end of the study period)	Change in overall suicide and suicide by other methods (reported until the end of the study period)	IRR (95% CI)	Economic and social factors, if reported in the study
**Pesticides in high-income countries**					
England, Wales, and Scotland^15^	1972: Occupational license required to purchase concentrated paraquat	Increasing trend in paraquat suicides: 5-10 cases in 1971; 30 cases in 1977	Not reported	Not suitable for calculation	Not reported
Ireland^16^	1968: Paraquat upscheduled into a poison 1975: Occupational license required to purchase paraquat	Suggested increasing trend in paraquat suicides: 7 cases in 1974; 13 cases in 1976	Not reported	Not suitable for calculation	Not reported
Germany^17^	1984 (Inferred): withdrawal of chlorinated hydrocarbons	Pesticide suicides almost eliminated: 6.4 per million in 1983; 0.5 per million in 2010	Overall suicide rate decreased: 23.6/100 000 in 1983; 12.3/100 000 in 2010. Other methods also decreased.	Pesticide: 0.42 (0.36-0.49) Other methods: 0.79 (0.78-0.81)	Reunification in 1990
Finland^18^	1960: Occupational license required for parathion	Parathion suicides decreased: 1.71/100 000 in 1959; 0.32/100 000 in 1973	No change in overall suicide from 1959 to 1965, and then an increase: 20.6/100 000 in 1959; 24.5/100 000 in 1973	Parathion: 0.37 (0.24-0.59) Other methods: 1.01 (0.92-1.12)	Not reported
Crete (Greece)^19^	2003: Withdrawal of organophosphates (parathion; monocrotophos)	No change in pesticide suicides: 1.4/100 000 in 2002; 1.2/100 000 in 2007	No change in overall suicide: 37 cases in 2002; 37 in 2007	Pesticide: 0.86 (0.31-2.36) Other methods: 1.01 (0.61-1.68)	Not reported
Marseille (France) and overseas territories^20^	2007: Paraquat ban	9 Paraquat suicides in 2003-2007; 6 paraquat suicides in 2007-2011	Not reported	Not suitable for calculation	Also included some overseas territories, which may be less resourced
South Korea^21,22,23,24,25,26^	2011: Manufacture and import of paraquat prohibited 2012: Paraquat ban	49% Decrease in pesticide suicides: 5.26/100 000 in 2011; 2.67/100 000 in 2013^21^	13% Decrease in overall suicide: 34.9/100 000 in 2011; 30.26/100 000 in 2013.^21^ Suicide by carbon monoxide and medicines increased; suicide by hanging decreased.^21,25^	Pesticide: 0.51 (0.48-0.54)^21^ Other methods: 0.93 (0.91-0.95)^21^	Suicides increased after financial crisis in 2008^25^; decreasing or stable trends in rates of divorce, unemployment, and alcohol use^21^
Taiwan^12,27,28^	1980s-1990s: 36 Pesticide formulations were banned, most of which were organophosphates and carbamates	67% Decrease in pesticide suicides: 7.7/100 000 in 1987; 2.5/100 000 in 2010^27^	Decrease in overall suicide from 1983 to 1993, and then an increase: 18.8/100 000 in 1987; 21.3/100 000 in 2010^27^	Not suitable for calculation	Economic changes and increased unemployment in 1990s^28^
**Pesticides in low- and middle-income countries**					
Hungary^29^	Unspecified: “decreased access to highly toxic pesticides”	Decreasing trend in pesticide suicides: 312 cases in 1990; 75 cases in 2001	27% Decrease in overall suicide: 39.8/100 000 in 1990; 29.2/100 000 in 2001. Other suicide methods also decreased.	Not suitable for calculation	Upper-middle income country
Inner Mongolia (China)^30^	2011: Organophosphates banned 2012: Paraquat banned	49% Decrease in pesticide suicides: 3.45/100 000 in 2008-2011; 1.75/100 000 in 2012-2015	33% Decrease in overall suicide: 7.2/100 000 in 2008-2011; 4.8/100 000 in 2012-2015	Pesticide: 0.51 (0.44-0.59) Other methods: 1.03 (0.89-1.20)	Upper-middle income country; social policies to alleviate poverty and fund public services
Sri Lanka^31,32,33^	1984: Parathion and methyl parathion banned 1995: WHO class 1 pesticides restricted 1998: Endosulfan banned	55% Decrease in pesticide suicides: 10.1/100 000 in 1996; 4.5/100 000 in 2009.^32^ Poisoning suicides also decreased.^31^	56% Decrease in overall suicide: 52.85/100 000 in 1995; 23.5/100 000 in 2005.^31^ Slight increase in suicides by hanging.^31^	Not suitable for calculation (rates for pesticide suicide only available for 1996 and 2009)	Secular trends in unemployment, alcohol misuse, divorce, and civil war unlikely to be associated with suicide trends^31,33^
Sri Lanka^34^	2008-2010: Dimethoate and fenthion banned 2009-2011: Paraquat banned	51% Decrease in pesticide suicides: 8.5/100 000 in 2011; 4.2/100 000 in 2015	21% Decrease in overall suicide: 18.3/100 000 in 2011; 14.3/100 000 in 2015	Pesticide: 0.46 (0.43-0.49) Other methods: 0.98 (0.92-1.04)	Secular trends in unemployment, alcohol misuse, divorce, and civil war unlikely to be associated with suicide trends^34^
India^35^	Unspecified: some WHO class 1 pesticides banned, including aldicarb and calcium cyanide	48% Decrease in pesticide suicides: 2.2/100 000 in 2001; 1.15/100 000 in 2014	Overall suicide rate not reported but likely to be unchanged or increasing as suicide by hanging increased rapidly	Not suitable for calculation (no intervention date)	Method of suicide varied in areas by level of economic development
Bangladesh^36^	2000: Some WHO class 1 pesticides banned, including organophosphates	65% Decrease in pesticide suicide: 6.3/100 000 in 1996; 2.2/100 000 in 2014	25% Decline in “overall unnatural deaths” from 1996-2014; no statistics provided for overall suicide; slight increase in suicide by hanging	Pesticide: 0.69 (0.66-0.71); other methods could not be calculated.	No significant change in unemployment, alcohol misuse, or divorce
**Domestic gas in high-income countries**					
England and Wales^37,38,39,40,41^	1958: Domestic gas detoxification	85% Decrease in domestic gas suicides: 10.4/100,000 in 1962-1963; 1.75/100 000 in 1970-1971^37^	33% Decrease in overall suicide: 24.15/100 000 in 1962-1963; 16.15/100 000 in 1970-1971^37^; some increase in overdose suicides^39^	Domestic gas: 0.07 (0.06-0.09)^40^ Other methods: 1.28 (1.22-1.34)^40^	Some increase in unemployment; was difficult to determine its effect^38^
Birmingham (England)^42^	1960s: Domestic gas detoxification	Decrease in domestic gas suicide: 87 cases in 1962; 12 cases in 1970	Decrease in overall suicide: 144 cases in 1963; 64 cases in 1970	Not suitable for calculation (no intervention date)	Not reported
Scotland^40,43^	1963: Domestic gas detoxification	95% Decrease in domestic gas suicide: 4.21/100 000 in 1962; 0.21/100 000 in 1975	10% Decrease in overall suicides: 9.10/100 000 in 1962; 8.16/100 000 in 1975	Domestic gas: 0.10 (0.06-0.15)^40^ Other methods: 1.57 (1.34-1.84)^40^	Not reported
Northern Ireland^44^	1964: Domestic gas detoxification	Domestic gas suicides almost eliminated: 2.06/100 000 in 1964; 0/100 000 in 1988	Decrease in overall suicide from 1964 to 1973 and then an increase: 5.42/100 000 in 1964; 9.69/100 000 in 1988	Domestic gas: 0.19 (0.08-0.47) Other methods: 1.24 (0.82-1.89)	Brief mention of civil disorders
Netherlands^40^	1963: Domestic gas detoxification	Domestic gas suicides almost eliminated: 14.0/million in 1962; 0.4/million in 1973	31% Increase in overall suicide: 65.63/million in 1962; 86.22/million in 1973	Domestic gas: 0.03 (0.01-0.06) Other methods: 1.66 (1.51-1.83)	Not reported
Minors (19 y or younger) in Vienna (Austria)^45^	1965: Domestic gas detoxification	Decrease in domestic gas suicides: 92 cases in 1956-1965; none after 1975	Decreasing trend in overall suicide: 8.4/100 000 in 1953-1962; 3.7/100 000 in 1993-2002	Domestic gas: 0.09 (0.04-0.18) Other methods: 0.87 (0.64-1.17)	Not reported
West Germany^46^	1963: Domestic gas detoxification	97% Decrease in domestic gas suicides: 1963-1976 (2.24 to 0.07/100 000)	12% Increase in overall suicide: 19.4/100 000 in 1963; 21.7/100 000 in 1976; decreasing trend from 1978 to 1989	Domestic gas: 0.03 (0.02-0.04) Other methods: 1.26 (1.23-1.29)	Not reported
Belgium^47^	1966: Domestic gas detoxification	87% Decrease in domestic gas suicides: 0.7/100 000 in 1968-1972; 0.09/100 000 in 1978-1981	34% Increase in overall suicide: 15.75/100 000 in 1968-1972; 21.07/100 000 in 1978-1981	Domestic gas: 0.13 (0.09-0.18) Other methods: 1.39 (1.35-1.44)	Not reported
Switzerland^48^	1955: Domestic gas detoxification	52% Decrease in domestic gas suicides: 3.6/100 000 in 1954; 1.72/100 000 in 1965	18% Decrease in overall suicide: 22.61/100 000 in 1954; 18.56/100 000 in 1965	Domestic gas: 0.48 (0.37-0.61) Other methods: 0.89 (0.81-0.97)	Not reported
Japan^49^	Early 1970s: domestic gas detoxification	42% Decrease in domestic gas suicides: 1.12/100 000 in 1969; 0.65/100 000 in 1982	20% Increase in overall suicide: 14.55/100 000 in 1969; 17.51/100 000 in 1982	Domestic gas: 0.82 (0.75-0.89) Other methods: 1.25 (1.22-1.27)	Not reported
US^50^	1940s-1950s: Domestic gas detoxification	Domestic gas suicides almost eliminated: 0.73/100 000 in 1950; 0.02/100 000 in 1970	Slight decrease in overall suicide from 1950 to 1960 and then an increase: 11.26/100 000 in 1950; 11.45/100 000 in 1970	Domestic gas: 0.14 (0.12-0.16) Other methods: 0.99 (0.97-1.01)	Not reported
Australia^51^	Early to mid-1960s: Domestic gas detoxification	58% Decrease in domestic gas suicides: 1.33/100 000 in 1960; 0.55/100 000 in 1970	17% Increase in overall suicide: 10.03/100 000 in 1960; 11.7/100 000 in 1970; continued increasing in 1990	Domestic gas: 0.42 (0.31-0.55) Other methods: 1.28 (1.18-1.39)	Not reported
**Motor vehicle exhaust in high-income countries**					
England and Wales^41,52,53^	1993: Catalytic converters	58% Decrease in motor exhaust suicides: 2.54/100 000 in 1992; 1.06/100 000 in 1998^52^	8% Decrease in overall suicide: 10.69/100 000 in 1992; 9.83/100 000 in 1998^52^	Motor exhaust: 0.42 (0.38-0.46)^52^ Other methods: 1.08 (1.03-1.12)^52^	Not reported
Scotland^53,54^	1993: Catalytic converters	73% Decrease in motor exhaust suicides: 2.11/100 000 in 1992; 0.57/100 000 in 2003^54^	Overall suicide increases and then returns to same rate in 2003; some increase in hanging	Motor exhaust: 0.27 (0.18-0.41)^54^ Other methods: 1.13 (1.02-1.25)^54^	Not reported
Switzerland^55^	1986: Catalytic converters	61% Decrease in motor exhaust suicides: 1.98/100 000 in 1985; 0.78/100 000 in 1995; continued decreasing in 2005	14% Decreasing trend in overall suicide: 23.75/100 000 in 1985; 20.35/100 000 in 1995; continued decreasing in 2005	Motor exhaust: 0.39 (0.29-0.54) Other methods: 0.90 (0.83-0.97)	Not reported
Japan^56^	1975: Motor emission standards to partially reduce carbon monoxide (still fatal at 4.5%)	106% Increase in motor exhaust suicides: 0.6/100 000 in 1974; 1.24/100 000 in 1981	No change in overall suicides: 17.5/100 000 in 1974; 17.28/100 000 in 1981	Motor exhaust: 2.07 (1.89-2.27) Other methods: 0.95 (0.93-0.97)	Not reported
US^57^	1968: Motor emission standards to partially reduce carbon monoxide	Motor exhaust suicides decreased in correlation with ownership rates of older cars	Not reported	Not suitable for calculation	Not reported
US^58,59^	1975: Catalytic converters	70% Decrease in motor exhaust suicides from 1975 to 2010^59^	Not reported	Motor exhaust: 1.05 (could not be calculated as no case numbers were available)^58^	Improved medical care for carbon monoxide exposure
Australia^60^	1986: Catalytic converters	Increase in motor exhaust suicides: 2.1/100 000 in 1985; 2.85/100 000 in 1995	Not reported	Motor exhaust: 1.36 (1.18-1.56)	Not reported
**Medicines in high-income countries**					
England and Wales^61,62,63^	1998: Pack size limit of paracetamol and salicylates (32 tablets in pharmacies; 16 in other retail)	43% Reduction in paracetamol suicides: 149 cases in 1997; 69 cases in 2009^62^; salicylate deaths also decreased	Decreasing trend in overall suicide: 4830 cases in 1997; 4682 cases in 2009^62^; deaths by other medicines and poison also decreased^61^	Paracetamol: 0.60 (0.46-0.77)^62^ Other methods: 0.98 (0.94-1.02)^62^	Not reported
England and Wales^64,65^	2005-2007: Phased withdrawal of paracetamol + dextropropoxyphene (co-proxamol)	61% Reduction in paracetamol + dextropropoxyphene suicides from 1998-2004 to 2005-2010^65^	Some decrease in overall suicide but not statistically significant: 4883 cases in 2004; 4528 cases in 2010^65^	Dextropropoxyphene: 0.04 (0.02-0.08)^65^ Other methods: 0.92 (0.88-0.96)^65^	Recession in 2008
Scotland^66^	2005-2007: Phased withdrawal of paracetamol + dextropropoxyphene (co-proxamol)	Decrease in annual paracetamol + dextropropoxyphene deaths: 37 in 2000-2004; 10 in 2006	Not reported; no change in suicide by other analgesics	Not suitable for calculation (unclear if the deaths were all suicides)	Not reported
Florida (US)^67^	2010: Withdrawal of propoxyphene	Decrease in propoxyphene suicides: 155 cases in 2008-2010; 22 cases in 2010-2012	Not reported	Not suitable for calculation (unclear if propoxyphene was always the cause of death)	Not reported
Sweden^68^	1985: Withdrawal of barbiturates (except phenobarbitone for epilepsy)	83% Decrease in barbiturate suicides: 1.15/100 000 in 1984; 0.2/100 000 in 1990	13% Decrease in overall suicide: 30.3/100 000 in 1969; 25.9/100 000 in 1992; some increase in suicide by analgesics and antidepressants	Barbiturates: 0.17 (0.10-0.29) Other methods: 0.89 (0.84-0.94)	Not reported
Japan^69^	1961: Upscheduled barbiturates from over-the-counter to prescription only	Sedative suicides almost eliminated: 5.6/100 000 in 1960; close to 0 in 1980	Decrease in overall suicide from 1960-1966; overall suicide and other methods increased after 1970	Sedatives: 0.22 (0.21-0.23) Not sedatives: 0.86 (0.84-0.88)	Not reported
Australia^70^	1967: Pack size limit of barbiturates (from 50 tablets and 2 repeats to 25 tablets only)	28% Decrease in drug-related suicides: 10.1/100 000 in 1966; 7.31/100 000 in 1970	18% Decrease in overall suicide: 15.19/100 000 in 1966; 12.38/100 000 in 1970	Drugs: 0.72 (0.65-0.80) Not drugs: 1.00 (0.87-1.14)	Not reported
Sweden^71^	2004: Pack size limit of caffeine tablets (from 250 tablets to 30 tablets)	12 Suicides with caffeine as the cause of death from 1994-2000; 0 after 2007	Not reported	NA	Not reported
**Combined interventions**					
Hradec Králové (Czech Republic)^72^	1970s: Catalytic converters 1990-1995: Domestic gas detoxification	Annual carbon monoxide suicide cases decrease from 1970-1979 to 1990-1999	Annual suicide cases decrease from 1970-1979 to 1990-1999	Not suitable for calculation (was difficult to characterize carbon monoxide deaths)	Upper-middle income country; higher suicide risk for areas with lower socioeconomic profile
Denmark^73,74^	1980s: Domestic gas detoxification 1986: Restrictions on dextropropoxyphene and barbiturate prescribing 1989: Catalytic converters	1985-2000: Domestic gas suicides almost eliminated; 64% decrease in motor exhaust suicides; 65% decrease in analgesic suicides; barbiturate suicides almost eliminated	54% Decrease in overall suicide: 27.89/100 000 in 1985; 13.72/100 000 in 2000; multiple co-interventions at the same time likely contributed to reduced incidence of suicide by other methods	Domestic gas: 0.41 (0.24-0.70) Motor exhaust: 0.43 (0.32-0.58) Analgesics: 0.88 (0.67-1.17) Barbiturates: 0.29 (0.17-0.50)	High-income country
Brisbane (Australia)^75^	1967: Domestic gas detoxification 1967: Pack size limit of barbiturates	Carbon monoxide suicides and barbiturate overdoses declined after 1967	44% Decrease in overall suicide rate: 34.95/100 000 in 1966; 19.7/100 000 in 1973	NA	High-income country

Abbreviations: IRR, incidence rate ratio; NA, not applicable; WHO, World Health Organization.

^a^ IRRs (95% CIs) were calculated using the annual data at a set number of years (1 year before intervention, 5 years after intervention for pesticides and medicines, or 10 years after intervention for gases). The World Bank historical classification was used for the income level of each country at the time of intervention, if available. If unavailable, the classification in 1987 was the earliest possible reference.^76^

### Statistical Analysis

We extracted data, when available, on suicide rates before and after a clearly defined intervention for a standardized before-and-after comparison. Because each study had different periods, we used annual data from 1 year before the intervention compared with 5 (for liquid or solid poison) or 10 (for gases) years after the intervention. Gases were evaluated after a longer time to account for regional differences and slow changes. Natural gas generally replaced coal gas sources within 10 years, whereas most older cars that predated legislative requirements would retire within 10 years in high-income countries.^44,60^

We used a preintervention time point 1 year before the intervention given that the suicide rate during the year of the intervention could be erratic depending on when and how the intervention was implemented. If data for these time points were unavailable, we used the closest years available. If multiple studies examined the same intervention, we used the study with the most detailed quantitative data.

Incidence rate ratio (IRR) was calculated as the incidence rate of suicides (usually number of suicides per 100 000 people) after the intervention divided by incidence rate before the intervention. The SE was calculated by adding the reciprocal of annual cases before and after the intervention, and CIs were calculated using log_e_ (IRR) and SE (eMethods 2 in the Supplement). If the number of annual cases of suicide was not available, we retrieved data from global websites, such as the World Bank,^78^ for a population estimate to calculate CIs. We used ImageJ, version 1.53e (National Institutes of Health) to quantify any data that were presented in graphical form only. We calculated IRRs for suicide by the restricted poison and by other methods of suicide (any method that was not the restricted poison), and then we graphed IRRs on forest plots on a log scale to show the relative change in suicide rates before and after the intervention.

We combined the same incidence rate data to quantify the change in suicide rate per 100 000 people before and after the intervention. These data were graphed on a scatterplot with linear regression to examine the association between the change in suicide by targeted poison and the change in suicide by other methods. Evidence for method substitution would be shown if there was an inverse association (eg, a slope of −1 would indicate that decreases in suicide by the restricted poison was, on average, matched by an equal but opposite change in other methods of suicide). We also graphed a scatterplot with linear regression to examine changes in suicide by other methods over time based on the year of the intervention. In addition, we calculated the median change in suicide by the restricted poison and suicide by other methods.

We repeated all analyses with overall suicide rates instead of suicide by other methods (eFigures 4-9 in the Supplement). We did not conduct a meta-analysis because the studies were heterogeneous with different interventions, dates, and locations. All graphs and analyses were produced using GraphPad Prism, version 9.1 (GraphPad).

## Results

The database searches retrieved 12 557 studies, of which 4916 were duplicates. Following the PRISMA guidelines, we performed title and abstract screening on 7657 studies, leading to the exclusion of 7566 studies. We reviewed 91 full-text articles, and 62 studies^12,15,16,17,18,19,20,21,22,23,24,25,26,27,28,29,30,31,32,33,34,35,36,37,38,39,40,41,42,43,44,45,46,47,48,49,50,51,52,53,54,55,56,57,58,59,60,61,62,63,64,65,66,67,68,69,70,71,72,73,74,75^ from 26 countries met the eligibility criteria for inclusion (eFigure 1 in the Supplement). Restricted substances included pesticides (15 countries), domestic gas (14 countries), motor vehicle exhaust (9 countries), and pharmaceuticals (8 countries). Different strategies to restrict access to poison included banning or withdrawing them from the market, reducing concentration, limiting the quantity sold, and allowing access for only a specific occupation or medical condition.

The studies investigated interventions in Europe, the US, Australia, and Asia, with multiple studies on all poison classes in the United Kingdom (eFigure 2 in the Supplement). We found no research from South America and Africa. Most studies were conducted in high-income countries,^12,15,16,17,18,19,20,21,22,23,24,25,26,27,28,37,38,39,40,41,42,43,44,45,46,47,48,49,50,51,52,53,54,55,56,57,58,59,60,61,62,63,64,65,66,67,68,69,70,71,73,74,75^ with only 9 studies on pesticide and gas restrictions in low- and middle-income countries.^29,30,31,32,33,34,35,36,72^

We evaluated each study using the ROBINS-I tool and found that most studies were prone to at least some low to medium risk of bias. Studies with limited trend analysis or limited preintervention data were at higher risk of confounding bias.^15,16,17,19,20,26,29,42,44,46,47,53,66,71^ These risks of bias are presented in a heat map (eTable 1 in the Supplement).

### Pesticide Restrictions

A total of 23 studies reported pesticide interventions in 15 countries (Table).^12,15,16,17,18,19,20,21,22,23,24,25,26,27,28,29,30,31,32,33,34,35,36^ Occupational licensing for conditional access to pesticides generally occurred earlier (1960-1975) than complete bans (1984-2012). Specific pesticides and classes that were restricted included organophosphates, parathion, organochlorines, and paraquat.

**Figure 1.**
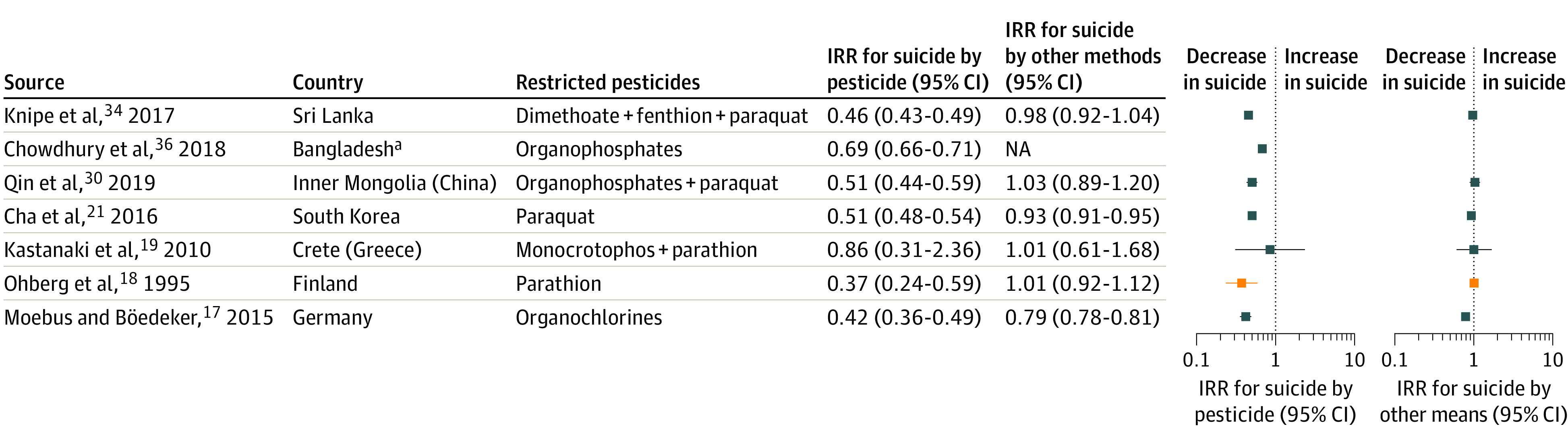
Forest Plots for Changes in Suicide by Pesticide and Suicide by Other Methods Blue squares indicate ban of pesticide; NA, not applicable; orange squares, occupational license. ^a^Data on overall suicide or other methods of suicide were not available.

The incidence of suicides by pesticide decreased in most studies (19 of 23).^12,17,18,21,22,23,24,25,26,27,28,29,30,31,32,33,34,35,36^ Seven interventions in 7 countries were suitable for IRR calculations, with 6 countries showing reduced incidence of suicides by pesticide (Figure 1).^17,18,19,21,30,34,36^ Five interventions were associated with decreases in suicides by pesticide were reported in Germany, Finland, South Korea, inner Mongolia, Sri Lanka, and Bangladesh, with IRRs ranging from 0.37 to 0.69.^17,18,21,30,34^ The IRRs for suicide by other methods suggested a decrease in other methods in Germany (0.79; 95% CI, 0.78-0.81) and South Korea (0.93; 95% CI, 0.91-0.95). Suicide by other methods did not change in Finland, Crete (Greece), Inner Mongolia, and Sri Lanka.^17,18,19,21,30,34^ Suicide by other methods was not reported in Bangladesh, but there was a “25% decline in unnatural deaths.”^36^^(pp175,178)^

In Sri Lanka, earlier pesticide bans from 1984 to 1998 included parathion and endosulfan. The bans were associated with a decrease in suicides by poisoning, with 35 of 100 000 suicides in 1984 and 20 of 100 000 suicides in 2000.^31,33,34^ In Taiwan, various pesticide formulations were banned from 1980. Suicides by pesticide decreased by 67% from 1987 to 2010, whereas the overall suicide rate and suicide by other methods gradually increased after 1993.^12,27,28^

Other studies included occupational licensing for paraquat in Ireland and the United Kingdom (England, Wales, and Scotland), and a paraquat ban in Marseille, France.^15,16,20^ The sample sizes in these studies were too small to enable us to calculate changes before and after the intervention. Studies in India and Hungary did not specify the date of pesticide interventions but reported a decline in suicides by pesticide over time.^29,35^ However, the incidence of hanging increased rapidly in India and was associated with an overall increase in suicide rates.^35^

### Gas Restrictions

Detoxification of domestic gas refers to the gradual removal of carbon monoxide from gas lines or replacing coal gas with natural gas. This detoxification was implemented in 14 countries from the 1950s to the 1990s (Table).^37,38,39,40,41,42,43,44,45,46,47,48,49,50,51,72,73,74,75^

**Figure 2.**
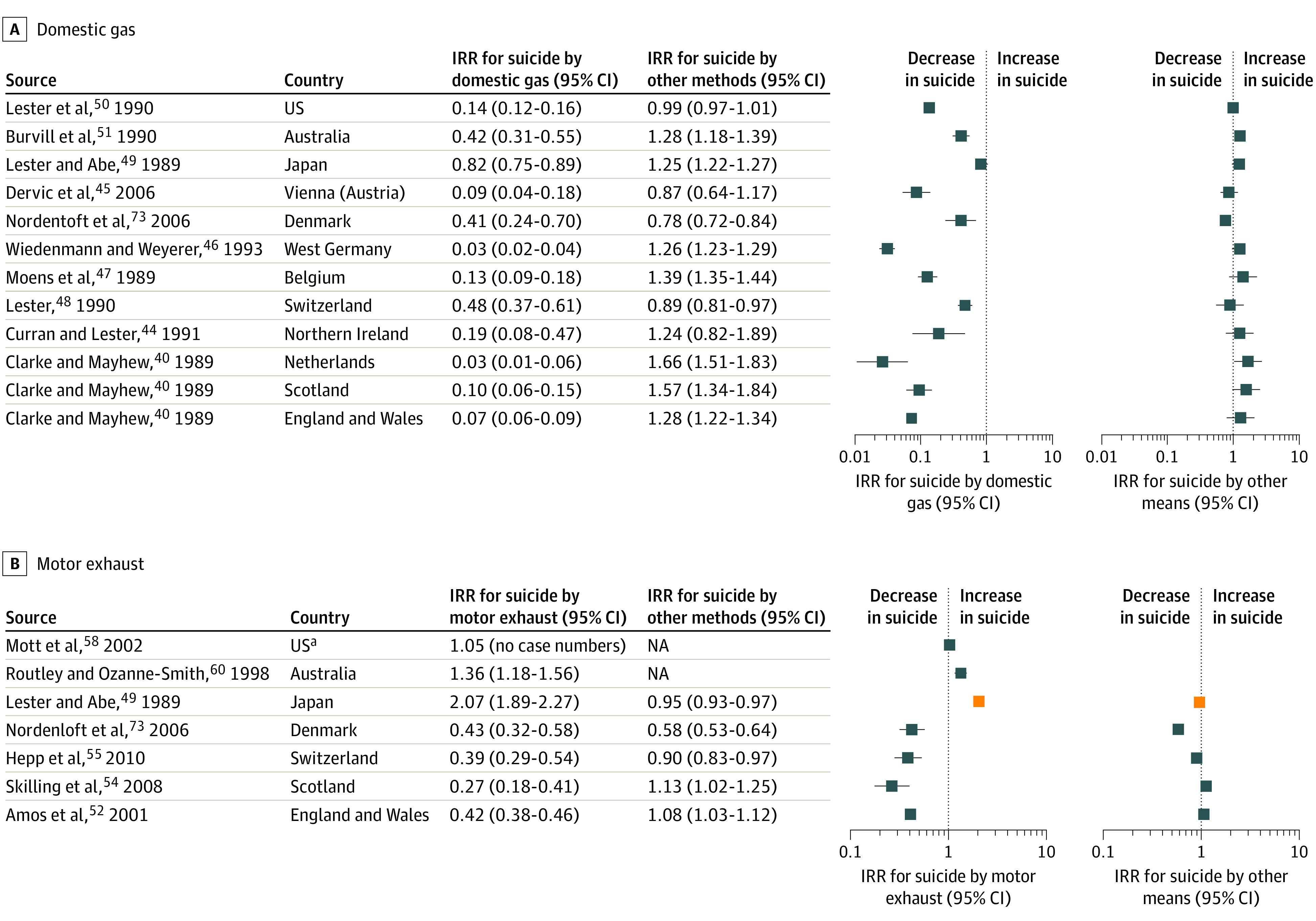
Forest Plots for Changes in Suicide by Domestic Gas and Motor Exhaust and Suicide by Other Methods In panel B, blue squares represent catalytic converter, and orange squares represent (4.5% emission) of carbon monoxide in cars. NA indicates not applicable. ^a^Data on overall suicide or other methods of suicide were not available.

Incidence rate ratios were calculated for 13 countries, which all reported reduced incidence of suicide by domestic gas (Figure 2). The largest decreases in domestic gas IRR were reported in the Netherlands (0.03; 95% CI, 0.01-0.06) and West Germany (0.03; 95% CI, 0.02-0.04).^40,46^

The incidence of suicide by other methods subsequently decreased in Switzerland (IRR, 0.89; 95% CI, 0.81-0.97) and Denmark (IRR, 0.78; 95% CI, 0.72-0.84)^48,73^ but did not change in the US,^50^ Vienna (Austria),^45^ and Northern Ireland.^44^ Suicide by other methods increased in 8 countries: England, Wales, Scotland, the Netherlands, Belgium, West Germany, Japan, and Australia, with IRRs ranging from 1.25 to 1.66.^40,46,47,49,51^

Catalytic converters and other motor emission legislations reduced carbon monoxide concentration in motor vehicle exhaust. This change was associated with a gradual decrease in carbon monoxide exposure risk because new cars were manufactured and purchased and older cars were removed from service. Nine countries reported suicides by motor exhaust from 1968 to 1993 (Table).^41,52,53,54,55,56,57,58,59,60,72,73,74^ Most countries introduced mandatory catalytic converters in new cars (carbon monoxide <0.5%).^53,57^ Japan’s motor legislation was more limited (carbon monoxide <4.5%), and the US reduced carbon monoxide emissions before mandating catalytic converters.^56,57^

Incidence rate ratio was calculated for 8 countries (Figure 2). Four interventions in 5 countries were associated with decreases in suicides by motor exhaust, with IRRs reported in England and Wales (0.42; 95% CI, 0.38-0.46), Scotland (0.27; 95% CI, 0.18-0.41), Switzerland (0.39; 95% CI, 0.29-0.54), and Denmark (0.43; 95% CI, 0.32-0.58).^52,54,55,73^ The incidence of suicides by motor exhaust was unlikely to have changed in the US (IRR, 1.05) or increased in Australia (IRR, 1.36; 95% CI, 1.18-1.56) and Japan (IRR, 2.07; 95% CI, 1.89-2.27).^56,58,60^ The incidence of suicide by other methods subsequently decreased in Switzerland, Denmark, and Japan, with IRRs ranging from 0.58 to 0.95, or increased slightly in England and Wales (IRR, 1.08; 95% CI, 1.03-1.12) and Scotland (IRR, 1.13; 95% CI, 1.02-1.25).

The incidence of suicides by carbon monoxide and overall suicides declined over time in Hradec Králové, Czech Republic. Domestic gas detoxification appeared to correspond to this trend more closely than catalytic converters.^72^

### Pharmaceutical Restrictions

Seven countries reported pharmaceutical interventions (Table).^61,62,63,64,65,66,67,68,69,70,71,73,74,75^ Targeted medicines included paracetamol, salicylates, dextropropoxyphene, barbiturates, and caffeine tablets. Strategies to limit access to medicine included limiting tablet pack size, withdrawal from market, therapeutic restriction by indication, and upscheduling (from over-the-counter to prescription drug only).

**Figure 3.**
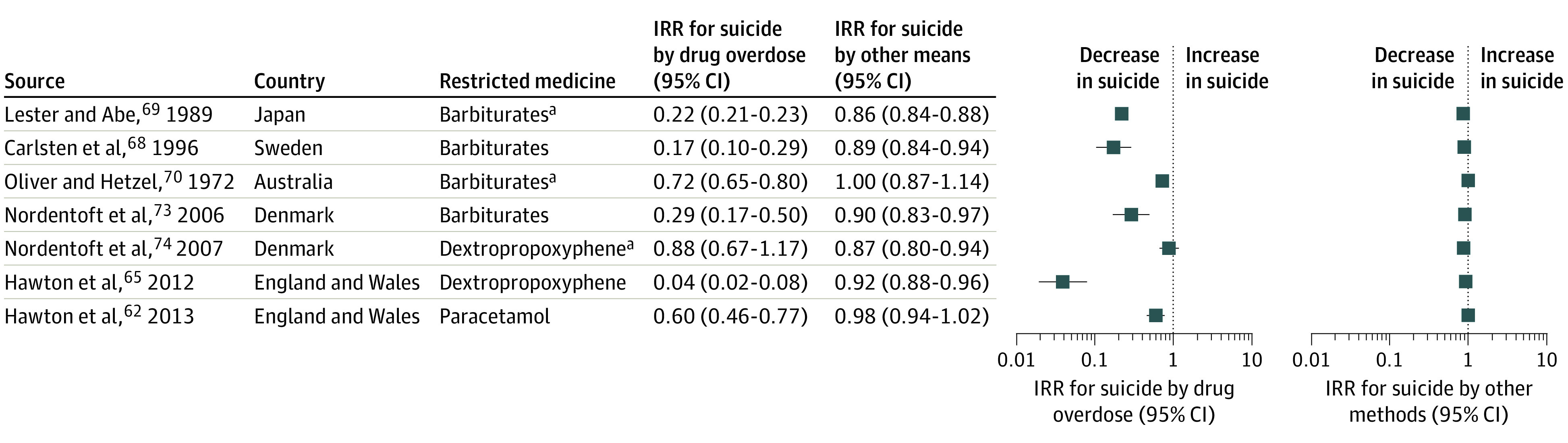
Forest Plots for Changes in Suicide by Drug Overdose and Suicide by Other Methods Barbiturates in Japan were up-scheduled to prescription-only access. Barbiturates in Sweden and Denmark and dextropropoxyphene in Denmark were placed under therapeutic restriction. Barbiturates in Australia and paracetamol in England and Wales had pack-size limits. Dextropropoxyphene in England and Wales was withdrawn from the market. ^a^Indicates studies that recorded suicide by a broader drug class (eg, analgesic and sedative).

Seven interventions in 6 countries were suitable for IRR calculations (Figure 3).^62,65,68,69,70,73,74^ Six interventions were followed by reduced incidence of suicides by medicine overdose, with the largest decrease after the dextropropoxyphene ban in England and Wales (IRR, 0.04; 95% CI, 0.02-0.08).^65^ Four barbiturate interventions were associated with decreased incidence of suicides by overdose, with IRRs reported in Denmark (0.29; 95% CI, 0.17-0.50), Australia (0.72; 95% CI, 0.65-0.80), Sweden (0.17; 95% CI, 0.10-0.29), and Japan (0.22; 95% CI, 0.21-0.23).^68,69,70,73^ Paracetamol pack size limits were also associated with a decrease in suicides by paracetamol overdose in England and Wales (IRR, 0.60; 95% CI, 0.46-0.77).^62^ Prescribing restrictions on dextropropoxyphene in Denmark did not appear to change the incidence of suicide by analgesic.^73,74^

The incidence of suicide by other methods decreased after barbiturates restrictions in Sweden, Japan, and Denmark, with IRRs ranging from 0.86 to 0.90, as well as dextropropoxyphene restrictions in Denmark (IRR, 0.87; 95% CI, 0.80-0.94) and England and Wales (IRR, 0.92; 95% CI, 0.88-0.96). Suicide by other methods did not appear to change after pack size limits of paracetamol in England and Wales and of barbiturates in Australia.

Other studies reported dextropropoxyphene withdrawal in Scotland and the US (Florida) as well as caffeine tablet pack size limits in Sweden. Suicides by dextropropoxyphene declined in Scotland after 2 years,^66^ whereas Florida reported a decreasing trend in suicides by dextropropoxyphene, and Sweden reported a decrease in suicides by caffeine intoxication.^67,71^

### Analysis for Method Substitution

The median (IQR) change in method-specific suicide rates after 29 interventions was −1.18 (−2.03 to −0.46) per 100 000 people during the same follow-up period (5 years for pesticides and medicines, and 10 years for gases). The change in other methods was distributed normally and centered around a median (IQR) of −0.09 (−2.22 to 1.65) per 100 000 people.

**Figure 4.**
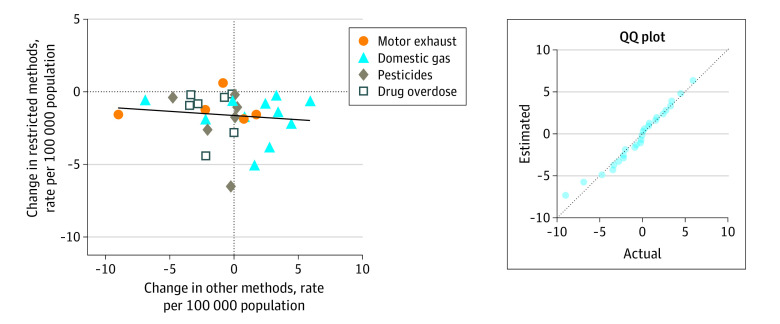
Scatterplot With Linear Regression of Change in Suicide by Restricted Methods vs Change in Suicide by Other Methods Line-of-fit metric *y* = −0.058x – 1.621 with x representing the change in suicide by other methods; linear regression slope = −0.06 (95% CI, −0.25 to 0.14). Inset, the quantile-quantile (QQ) plot shows a normal distribution.

No significant association was found (linear regression slope, −0.06; 95% CI, −0.25 to 0.14) between changes in suicide by the restricted poison and suicide by other methods (Figure 4). This result suggests that random variations or other unrelated factors were associated with the observed changes in other methods of suicide. Increases in other methods were often associated with domestic gas detoxification and earlier periods (1960-1975) (eFigure 3 in the Supplement). A conservative estimate of approximately 57 355 poison-specific suicides occurred annually before the interventions were implemented (eTable 2 in the Supplement); these cases may have benefited from means restrictions.

## Discussion

Overall, this synthesis of the literature suggests that means restriction of a range of poison was associated with lower incidences of suicide. We found no evidence to suggest that change in other suicide methods that coincided with bans and restrictions was anything other than coincidental. A decrease in specific poisoning did not promote the use of other equally lethal methods. An increase in other suicide methods tended to be partial, to be focused on a subgroup, or to occur over a long time.^10,13^

Individuals usually prefer a specific method during a suicidal crisis, and any decrease in method-specific suicide rates is beneficial.^8,13^ Firearm ownership in the US has been associated with 4.8-times higher odds of suicide, whereas a 10% decrease in firearm ownership has been associated with a 4.2% decline in suicide by firearm and 2.5% decline in overall suicide.^7,8^ Bridge barriers have been associated with an 86% reduction in suicides by jumping at sites with barriers, 44% increase in suicides by jumping at sites without barriers, or a 28% net decrease of all suicides by jumping.^9^

No significant association was found between changes in suicides by poisoning and suicides by other methods. It seems likely that background historical trends in other methods may explain the many cases in which investigators have concluded that no method substitution occurred. When coal gas was phased out, decreased incidences of suicides by domestic gas coincided with increased access to psychotropic medicines and increased car ownership.^3,39,44,52,55^ Psychotropic medicines (especially barbiturates and tricyclic antidepressants) and cars without catalytic converters were relatively toxic from the 1960s to the 1980s until they were replaced or restricted.^39,44,52,55,58,73^ Benzodiazepines and selective serotonin reuptake inhibitors that were much less toxic in overdose became the most common sedatives and antidepressants.^39,68,73,75,79^

Pesticide restrictions were complex, with different chemicals and formulations available. Outcomes varied depending on what other pesticides were available or banned in the same time frame. Pesticide bans were associated with fewer suicides by pesticide more often than occupational licensing.^12,15,16,17,18,21,22,23,24,25,26,27,28,30,31,32,33,34,35,36^

Domestic gas detoxification was associated with reduced suicides by carbon monoxide, whereas motor exhaust interventions were sometimes associated with increasing trends of suicides by carbon monoxide. This finding likely reflects the total coverage and relative speed with which household domestic gas could be detoxified. Catalytic converters were applied only to new cars and can lose effectiveness over time.^57^

Most strategies to limit access to medicine were associated with decreases in suicides by drug overdose. Medicine restrictions can also be affected by changes in prescribing patterns.^75^ Sedative and hypnotic overdoses were common in the twentieth century, whereas analgesics and opioids are currently associated with medicine poisoning. Means restriction of opioids other than dextropropoxyphene has not been studied to date. Dextropropoxyphene has direct cardiac toxic effects and thus should not be considered a typical opioid. Research on opioid policies and opioid suicide rates should be a high priority given the current epidemic of prescription opioid misuse in high-income countries.^65,67^

A total of 57 355 poison-specific suicides were reported before the interventions were implemented (eTable 2 in the Supplement). This population represents the cohort who may benefit the most from means restriction of poison, but this population does not account for studies that occur during different periods. Means restriction had the greatest public health benefit when the restricted suicide method was common and highly lethal. This result was seen in Sri Lanka, where multiple pesticide bans were associated with 19 800 fewer suicides (based on time-trend analysis of overall suicide rate) in 1996 to 2005 vs 1986 to 1995.^31^ In contrast, car exhaust in Japan remained lethal because carbon monoxide was only partially reduced and the increase in suicides by motor exhaust was associated with an increase in car ownership.^56^

The choice of suicide method at a population level is complex but often varies by socioeconomic profile.^35^ Some violent self-harm methods (such as hanging, jumping, and sharp-object injury) tend to have lower cost than poisoning. Hanging remained a common method of suicide, and the incidence of hanging sometimes increased over time.^18,24,30,31,35,36,41,44,47,52,74^ Hanging is highly lethal and difficult to target with means restriction, although means restriction has a place in specific settings, such as ligature restrictions in prisons and hospitals.^52^

Many interventions such as detoxification of domestic gas and withdrawal of dextropropoxyphene were likely to be widespread, but these interventions were evaluated only in a small number of countries. Cost may be a factor, such as low-income countries using older cars without working catalytic converters. Bans of hazardous pesticides in low- and middle-income countries are considered highly cost-effective and should remain a high priority.^80^

We evaluated each study with the ROBINS-I tool and found that some studies were prone to selection and reporting bias or with poorly specified interventions. Methods varied considerably, with repeated cross-sectional studies and studies with limited follow-up periods providing less reliable estimates. Most studies considered preintervention trends, but only 4 studies conducted an interrupted time series analysis.^62,63,64,65^ Interrupted time series analysis is a robust method for estimating preintervention trends and changes in slope to ascertain the magnitude of the intervention. Ideally, this method would be used for future means restriction studies.

### Strengths and Limitations

This study has some strengths. To our knowledge, no other systematic review has focused on all poisons, quantitatively comparing poison-specific suicide rates and suicide by other methods. We collected and analyzed data on method-specific suicide rates and suicide by other methods before and after 29 interventions. This method allowed a comparison of different interventions with the same unit (rate per 100 000 people), but it does not account for different periods, preintervention trends, and cointerventions. Preintervention trends can change the reported outcome, such as when physicians were encouraged to prescribe alternate analgesics ahead of the dextropropoxyphene withdrawal.^65,66,67^ Social and economic detriments to mental health or quality of life, such as poverty, employment, substance misuse, and conflict, also alter the rates of suicidality and are potential confounding factors that are difficult to explain. Means restriction of multiple substances simultaneously or means restriction combined with other suicide prevention policies could have a synergistic benefit for suicide rates. However, quantifying this advantage was beyond the scope of this systematic review.

This study also has some limitations. First, this systematic review did not identify any studies from South America and Africa, which may affect the generalizability of the findings. Second, we found low representation of low- and middle-income countries, despite most suicides occurring in these countries.^2^ This low representation may be attributed to the search strategy, which included English studies only and excluded reports and other articles that were not published in academic journals. Low- and middle-income countries may be less likely to publish studies on suicide given that underreporting and stigma are common.^35^

## Conclusions

Means restriction appeared to be associated with decreased suicide rates and had the greatest benefit when it restricted suicide methods that were common and highly lethal. Coincidental changes in other methods likely reflects the background trends in other methods, and the overall changes across all studies did not provide support for the speculation that restrictive interventions lead to method substitution. Suicide prevention strategies should be synergistic, targeting both individuals who are at risk and broad population-level policies, including means restriction.

## Notes

Supplement. **eMethods 1.** Search Strategy **eMethods 2.** IRR and CIs Were Calculated With the Following Formulae **eFigure 1.** PRISMA Flow Diagram of Study Selection **eFigure 2.** Bubble Map Showing the Location of Means Restriction Studies Globally, With a Zoomed Inset from Europe **eFigure 3.** Scatter Plot With Linear Regression of Change in Suicide by Other Methods Versus Year of the Intervention (Line of Fit Metric: Y = −0.05572*X + 109.8, Linear Regression Slope of −0.06 (95% CI −0.12 to 0.009)) **eFigure 4.** Forest Plot for Changes in Overall Suicide After Pesticide Restrictions **eFigure 5.** Forest Plot for Changes in Overall Suicide After Domestic Gas Detoxification **eFigure 6.** Forest Plot for Changes in Overall Suicide After Motor Exhaust Interventions **eFigure 7.** Forest Plot for Changes in Overall Suicide After Medicine Restrictions **eFigure 8.** Scatter Plot With Linear Regression of Change in Suicide by Restricted Methods Versus Change in Overall Suicide (Line of Fit Metric: Y = 0.1660*X - 1.254, Linear Regression Slope of 0 · 16, 95% [CI −0.01 to 0.34]). **eFigure 9.** Scatter Plot With Linear Regression of Change in Overall Suicide Versus Year of the Intervention (Line of Fit Metric: Y = −0.05165*X + 100.2, Linear Regression Slope of −0 · 05, [95% CI −0.12 to 0.02]) **eTable 1.** Risk of Bias Heat Map Based on the ROBINS I Risk of Bias Tool for Uncontrolled Before-After Studies (Includes Interrupted Time Series) **eTable 2.** Approximate or Estimated Number of Annual Poison-Specific Suicides Reported One Year Before Each Intervention (or Closest Year Available). **eMethods 3.** Excluded Papers With Reasons
eReferences
