# Supplementary material for: Association Between Means Restriction of Poison and Method-Specific Suicide Rates: A Systematic Review
Source: JAMA Health Forum. 2021 Oct 15;2(10):e213042. doi: 10.1001/jamahealthforum.2021.3042 (PMC8727039; doi:10.1001/jamahealthforum.2021.3042)
Supplement: Supplement. — eMethods 1. Search Strategy eMethods 2. IRR and CIs Were Calculated With the Following Formulae eFigure 1. PRISMA Flow Diagram of Study Selection eFigure 2. Bubble Map Showing the Location of Means Restriction Studies Globally, With a Zoomed Inset from Europe eFigure 3. Scatter Plot With Linear Regression of Change in Suicide by Other Methods Versus Year of the Intervention (Line of Fit Metric: Y = −0.05572*X + 109.8, Linear Regression Slope of −0.06 (95% CI −0.12 to 0.009)) eFigure 4. Forest Plot for Changes in Overall Suicide After Pesticide Restrictions eFigure 5. Forest Plot for Changes in Overall Suicide After Domestic Gas Detoxification eFigure 6. Forest Plot for Changes in Overall Suicide After Motor Exhaust Interventions eFigure 7. Forest Plot for Changes in Overall Suicide After Medicine Restrictions eFigure 8. Scatter Plot With Linear Regression of Change in Suicide by Restricted Methods Versus Change in Overall Suicide (Line of Fit Metric: Y = 0.1660*X - 1.254, Linear Regression Slope of 0 · 16, 95% [CI −0.01 to 0.34]). eFigure 9. Scatter Plot With Linear Regression of Change in Overall Suicide Versus Year of the Intervention (Line of Fit Metric: Y = −0.05165*X + 100.2, Linear Regression Slope of −0 · 05, [95% CI −0.12 to 0.02]) eTable 1. Risk of Bias Heat Map Based on the ROBINS I Risk of Bias Tool for Uncontrolled Before-After Studies (Includes Interrupted Time Series) eTable 2. Approximate or Estimated Number of Annual Poison-Specific Suicides Reported One Year Before Each Intervention (or Closest Year Available). eMethods 3. Excluded Papers With Reasons eReferences [file jamahealthforum-e213042-s001.pdf]

Supplementary Online Content

Lim JS, Buckley NA, Chitty KM, Moles RJ, Cairns R. Association between means restriction of poison and method-specific suicide rates: a systematic review. *JAMA Health Forum*. 2021;2(10):e213042. doi:10.1001/jamahealthforum.2021.3042

eMethods 1. Search Strategy

eMethods 2. IRR and CIs Were Calculated With the Following Formulae

eFigure 1. PRISMA Flow Diagram of Study Selection

eFigure 2. Bubble Map Showing the Location of Means Restriction Studies Globally, With a Zoomed Inset from Europe

eFigure 3. Scatter Plot With Linear Regression of Change in Suicide by Other Methods Versus Year of the Intervention (Line of Fit Metric: Y = -0.05572*X + 109.8, Linear Regression Slope of -0.06 (95% CI -0.12 to 0.009))

eFigure 4. Forest Plot for Changes in Overall Suicide After Pesticide Restrictions

eFigure 5. Forest Plot for Changes in Overall Suicide After Domestic Gas Detoxification

eFigure 6. Forest Plot for Changes in Overall Suicide After Motor Exhaust Interventions

eFigure 7. Forest Plot for Changes in Overall Suicide After Medicine Restrictions

eFigure 8. Scatter Plot With Linear Regression of Change in Suicide by Restricted Methods Versus Change in Overall Suicide (Line of Fit Metric: Y = 0.1660*X - 1.254, Linear Regression Slope of 0·16, 95% [CI - 0.01 to 0.34]).

eFigure 9. Scatter Plot With Linear Regression of Change in Overall Suicide Versus Year of the Intervention (Line of Fit Metric: Y = -0.05165*X + 100.2, Linear Regression Slope of -0·05, [95% CI -0.12 to 0.02])

eTable 1. Risk of Bias Heat Map Based on the ROBINS I Risk of Bias Tool for Uncontrolled Before-After Studies (Includes Interrupted Time Series)

eTable 2. Approximate or Estimated Number of Annual Poison-Specific Suicides Reported One Year Before Each Intervention (or Closest Year Available).

Methods 3. Excluded Papers With Reasons

eReferences

This supplementary material has been provided by the authors to give readers additional information about their work.

© 2021 Lim JS et al. *JAMA Health Forum.*

eMethods 1. Search Strategy

Search terms used for Medline, Embase and PsycInfo (last searched 09/03/2020):

1 Suicide/

2 Suicide.mp.

3 1 or 2

4 Drug Overdose/

5 Poisoning/

6 Poison*.mp. [mp=title, abstract, original title, name of substance word, subject heading word, floating sub-heading word, keyword heading word, organism supplementary concept word, protocol supplementary concept word, rare disease supplementary concept word, unique identifier, synonyms]

7 Pesticides/

8 (Gas or charcoal or exhaust).mp. [mp=title, abstract, original title, name of substance word, subject heading word, floating sub-heading word, keyword heading word, organism supplementary concept word, protocol supplementary concept word, rare disease supplementary concept word, unique identifier, synonyms]

9 Acetaminophen/

10 Analgesics/ or Analgesics, Opioid/

11 Barbiturates/

12 paracetamol.mp.

13 (Medicine* or Medication*).mp. [mp=title, abstract, original title, name of substance word, subject heading word, floating sub-heading word, keyword heading word, organism supplementary concept word, protocol supplementary concept word, rare disease supplementary concept word, unique identifier, synonyms]

14 (Pharmacy or Pharmacies or Pharmacist or Pharmacists).mp. [mp=title, abstract, original title, name of substance word, subject heading word, floating sub-heading word, keyword heading word, organism supplementary concept word, protocol supplementary concept word, rare disease supplementary concept word, unique identifier, synonyms]

15 ((Domestic or household or house-hold) adj3 product*).mp.

16 ((Domestic or household or house-hold) adj3 chemical*).mp.

17 4 or 5 or 6 or 7 or 8 or 9 or 10 or 11 or 12 or 13 or 14 or 15 or 16

18 (Means Restriction or Restriction or Access or Withdraw* or Ban).mp. [mp=title, abstract, original title, name of substance word, subject heading word, floating sub-heading word, keyword heading word, organism supplementary concept word, protocol supplementary concept word, rare disease supplementary concept word, unique identifier, synonyms]

19 (Policy or Policies or Strateg* or Intervention* or Schedul* or Up-Schedul* or Upschedul* or Reschedul* or Re-Schedul*).mp. [mp=title, abstract, original title, name of substance word, subject heading word, floating sub-heading word, keyword heading word, organism supplementary concept word, protocol supplementary concept word, rare disease supplementary concept word, unique identifier, synonyms]

20 18 or 19

© 2021 Lim JS et al. *JAMA Health Forum.*

21 3 and 17 and 20

Search terms used for Scopus (last searched 09/03/2020):

1 Title-Abs-Key (suicide AND (overdose OR poison OR pesticide OR gas OR medication) AND (restriction OR prevention OR intervention))

Search terms used for Web of Science (last searched 09/03/2020):

1 TS=(suicide)

2 TS=(overdose OR poison OR pesticide OR gas OR medication)

3 TS=(restriction OR prevention OR intervention)

4 #1 and #2 and #3

© 2021 Lim JS et al. *JAMA Health Forum.*

eMethods 2. IRR and CIs Were Calculated With the Following Formulae[1]

IRR = Incidence Rate after intervention / Incidence Rate before intervention

Standard Error = $\sqrt{\left(\frac{1}{\text{annual cases before intervention}} + \frac{1}{\text{annual cases after intervention}}\right)}$

Lower bound = Ln (IRR) – (1.96 x Standard Error)

Upper bound = Ln (IRR) + (1.96 x Standard Error)

95% Confidence Interval of IRR = $(e^{\text{Lower bound}}, e^{\text{Upper bound}})$

© 2021 Lim JS et al. *JAMA Health Forum.*

eFigure 1. PRISMA Flow Diagram of Study Selection[2]

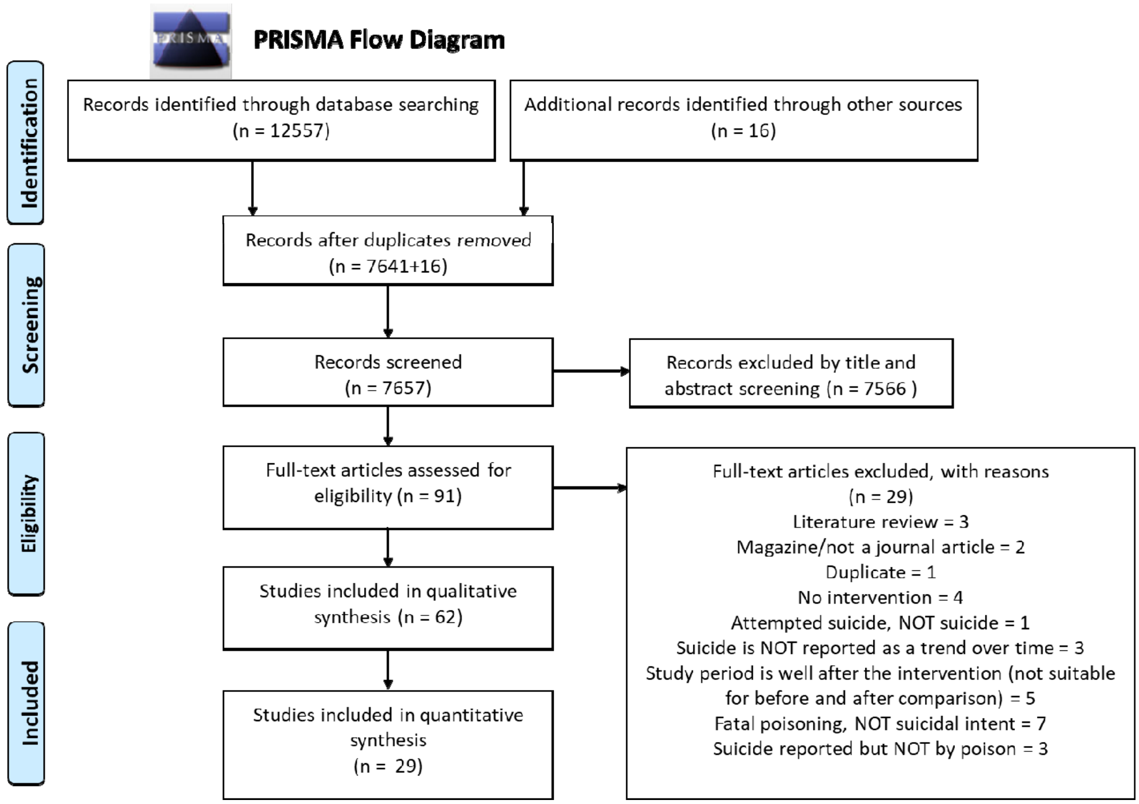

© 2021 Lim JS et al. *JAMA Health Forum.*

eFigure 2. Bubble Map Showing the Location of Means Restriction Studies Globally, With a Zoomed Inset from Europe

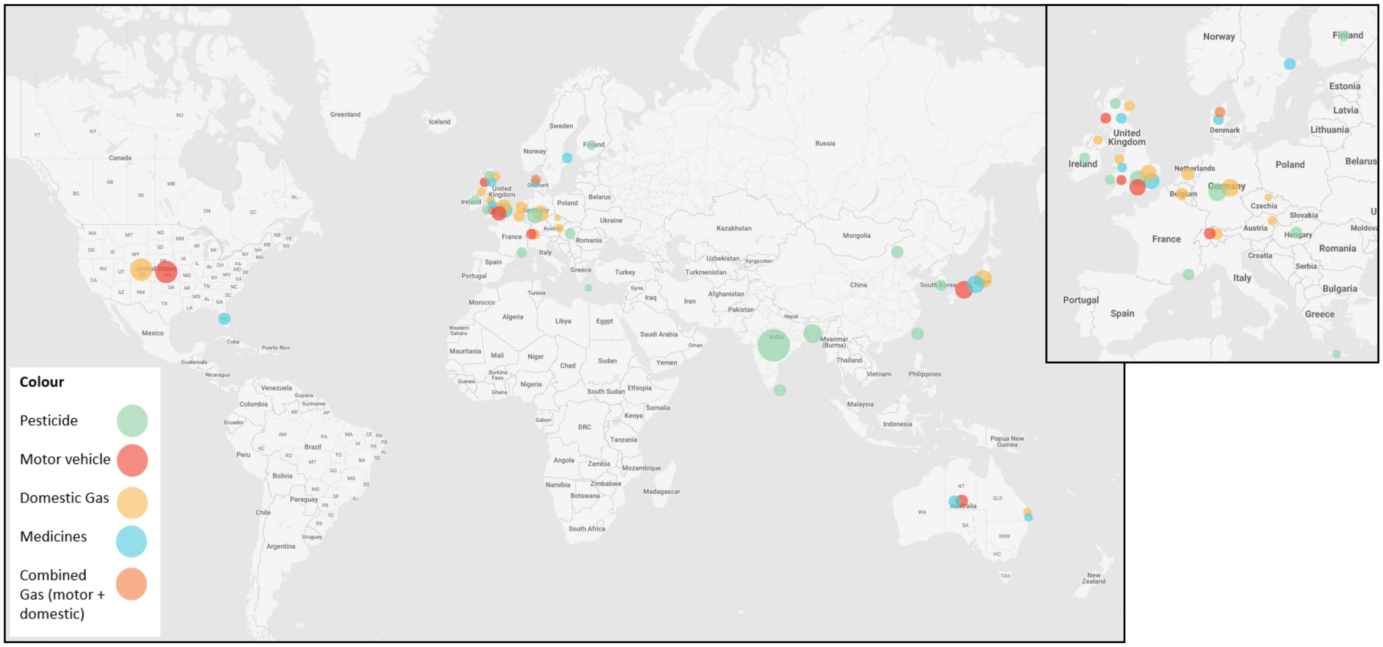

Colour refers to the type of poison, while bubble size is a rough visualisation of the study population size (bubble size is proportional to the cube root of the current population). Both regional and global populations have been plotted (e.g. there were both national and city-specific studies in Australia and USA)

© 2021 Lim JS et al. *JAMA Health Forum.*

eFigure 3. Scatter Plot With Linear Regression of Change in Suicide by Other Methods Versus Year of the Intervention (Line of Fit Metric: Y = -0.05572*X + 109.8 , Linear Regression Slope of -0.06 (95% CI -0.12 to 0.009))

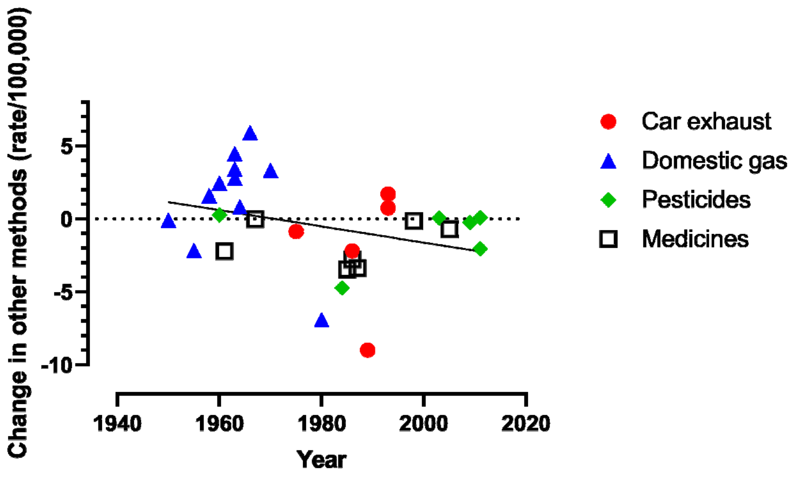

© 2021 Lim JS et al. *JAMA Health Forum.*

eFigure 4. Forest Plot for Changes in Overall Suicide After Pesticide Restrictions[3-9]

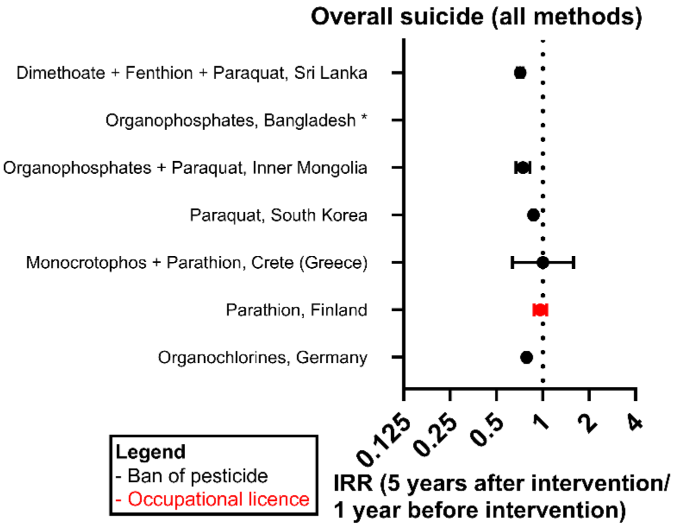

* data on overall suicide or other methods of suicide were not available

© 2021 Lim JS et al. *JAMA Health Forum.*

eFigure 5. Forest Plot for Changes in Overall Suicide After Domestic Gas Detoxification[10-20]

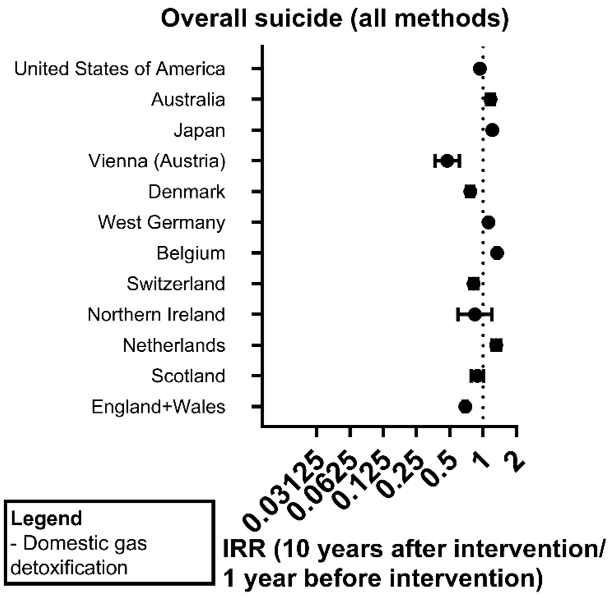

© 2021 Lim JS et al. *JAMA Health Forum.*

eFigure 6. Forest Plot for Changes in Overall Suicide After Motor Exhaust Interventions[19-26]

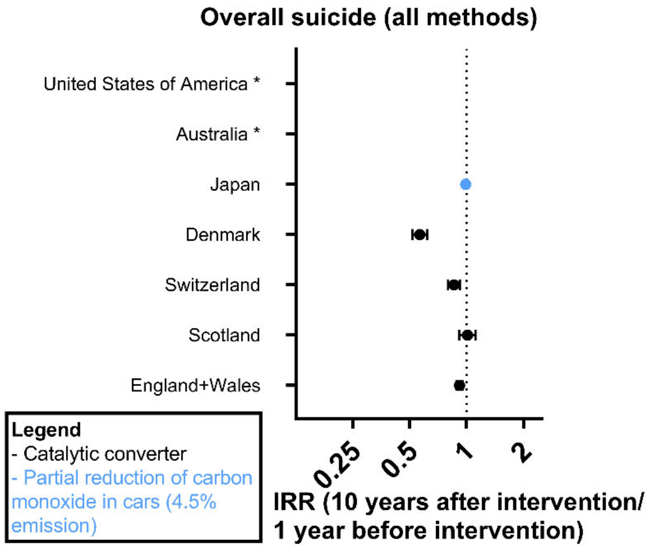

* data on overall suicide or other methods of suicide were not available

© 2021 Lim JS et al. *JAMA Health Forum.*

eFigure 7. Forest Plot for Changes in Overall Suicide After Medicine Restrictions[19,20,27-31]

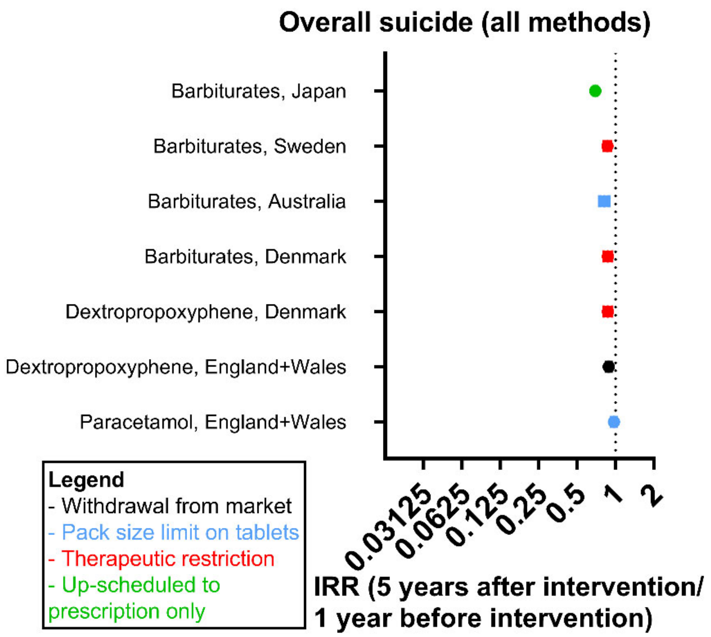

© 2021 Lim JS et al. *JAMA Health Forum.*

eFigure 8. Scatter Plot With Linear Regression of Change in Suicide by Restricted Methods Versus Change in Overall Suicide (Line of Fit Metric: Y = 0.1660*X - 1.254, Linear Regression Slope of 0·16, 95% [CI -0.01 to 0.34]).

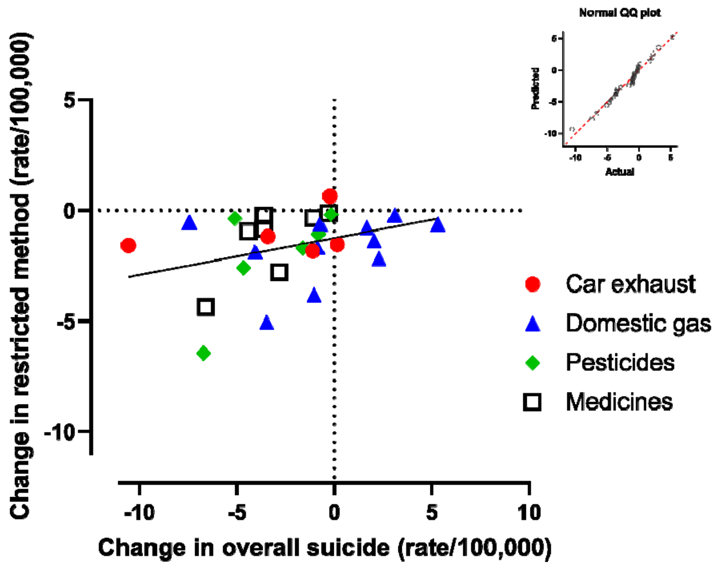

The QQ plot shows a normal distribution.

© 2021 Lim JS et al. *JAMA Health Forum.*

eFigure 9. Scatter Plot With Linear Regression of Change in Overall Suicide Versus Year of the Intervention (Line of Fit Metric: Y = -0.05165*X + 100.2, Linear Regression Slope of -0·05, [95% CI -0.12 to 0.02])

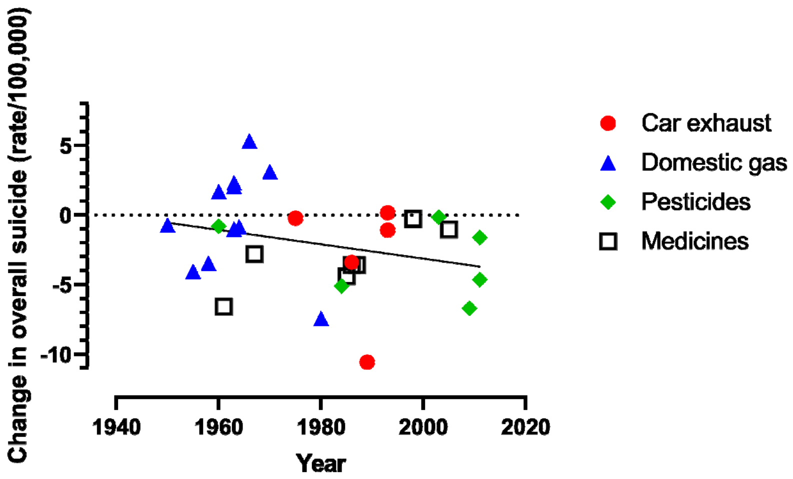

© 2021 Lim JS et al. *JAMA Health Forum.*

eTable 1. Risk of Bias Heat Map Based on the ROBINS I Risk of Bias Tool for Uncontrolled Before-After Studies (Includes Interrupted Time Series)[32]

Author	Location	Year of publica-tion	Risk of bias due to confounding	Selection bias of participants	Bias in classification of intervention	Bias due to deviations from intended interventions	Bias due to missing data	Bias in measurement of outcome	Selective reporting of outcome
Onyon [33]	England, Wales, Scotland	1987	Mid (limited trend analysis)	Low	Low	Mid-High (other changes included storage regulations and re-formulation)	High (missing data)	Mid (early data from Journal, later data from Office)	Low
Fitzgerald [34]	Ireland	1978	High (case numbers reported with no analysis)	Low	Low	Mid (paraquat licence difficult to enforce)	Low	Low	Mid (only 2 years follow-up after intervention)
Moebus [8]	Germany	2015	Mid (limited trend analysis)	Low	Mid (date of organochlorine intervention not clear but seems to be 1984)	Low-Mid (other pesticides restricted)	Low	Mid (administrative changes from East and West Germany to Germany)	Low
Ohberg [7]	Finland	1995	Low-Mid (some trend analysis)	Low	Low	Low	Low	Low	Low
Berecz [35]	Hungary	2005	High (case numbers reported with no analysis)	Low	High (no details of pesticide intervention)	Low-Mid (possible co-interventions such as treatment of depression)	Low	Low	High (repeated cross sectional)
Kastanaki [4]	Crete (Greece)	2010	Mid (limited trend analysis)	Mid (one island only)	Low	Mid (some interventions announced early)	Low	Low-Mid (coroners may not be consistent)	Low
Kervegant [36]	Marseille poison centre (France)	2013	High (case numbers reported with no analysis)	Mid (one poison centre only)	Low	Low	Low	Mid-High (overseas territories may have different procedures)	Low

© 2021 Lim JS et al. *JAMA Health Forum.*

Author	Location	Year of publica-tion	Risk of bias due to confounding	Selection bias of participants	Bias in classification of intervention	Bias due to deviations from intended interventions	Bias due to missing data	Bias in measurement of outcome	Selective reporting of outcome
Cha [9]	South Korea	2016	Low (did interrupted time series as sensitivity analysis)	Low	Low	Mid (paraquat purchase limited ahead of ban, other pesticides withdrawn)	Low	Low	Mid (only 2 years follow-up after intervention)
Cha [37]	South Korea	2019	Low-Mid	Low	Low	Mid (paraquat purchase limited ahead of ban, other pesticides withdrawn)	Low	Low	Mid (only 3 years follow-up after intervention)
Kim [38]	South Korea	2017	Low-Mid	Low	Low	Mid (paraquat purchase limited ahead of ban, other pesticides withdrawn)	Low	Low	Low-Mid (before and after study, missing 2011 and 2012)
Han [39]	South Korea	2018	Low-Mid	Low	Low	Mid (paraquat purchase limited ahead of ban, other pesticides withdrawn)	Low	Mid (only the top 5 causes of suicide are reported, this is 85% of suicides)	Low-Mid (only 4 years follow-up after intervention)
Myung [40]	South Korea	2015	Low-Mid	Low	Low	Mid (paraquat purchase limited ahead of ban, other pesticides withdrawn)	Low	Low	Mid (only 2 years follow-up after intervention)
Lee [41]	South Korea	2015	Mid-High (no pre-intervention analysis)	Low	Low	Mid (paraquat purchase limited ahead of ban, other pesticides withdrawn)	Low	Low	Mid (only 2 years follow-up after intervention)
Chang [42]	Taiwan	2012	Low-Mid	Low	Mid-High (multiple formulations restricted,)	Low	Mid (some missing data before 2002)	Low	Low

© 2021 Lim JS et al. *JAMA Health Forum.*

Author	Location	Year of publica-tion	Risk of bias due to confounding	Selection bias of participants	Bias in classification of intervention	Bias due to deviations from intended interventions	Bias due to missing data	Bias in measurement of outcome	Selective reporting of outcome
Lin [43]	Taiwan	2011	Low-Mid	Low	Mid-High (multiple formulations restricted)	Low	Low	Low	Mid-High (1993 was chosen when other methods did not increase)
Chen [44]	Taiwan	2013	Low-Mid	Low	Mid-High (multiple formulations restricted)	Low	Low	Low	Mid (reported poisoning deaths, not pesticide deaths)
Qin [6]	Inner Mongolia (China)	2019	Low-Mid	Low	Low	Low	Low	High (the increased number of collection centres/ increased cohort size may have influenced result)	Mid (only 3 years follow-up after intervention)
Gunnell [45]	Sri Lanka	2007	Low-Mid	Low	Low	Low	Low	Low	Mid (reported poisoning deaths, not pesticide deaths)
De Silva [46]	Sri Lanka	2012	Low-Mid	Low	Low	Low	Low	Low	Low-Mid (pesticide suicide rates were only provided for two years)
Knipe [47]	Sri Lanka	2014	Low-Mid	Low	Low	Low	Low	Low	Mid (reported poisoning deaths, not pesticide deaths)
Knipe [3]	Sri Lanka	2017	Low-Mid	Low	Low	Low	Low	Low	Low

© 2021 Lim JS et al. *JAMA Health Forum.*

Author	Location	Year of publica-tion	Risk of bias due to confounding	Selection bias of participants	Bias in classification of intervention	Bias due to deviations from intended interventions	Bias due to missing data	Bias in measurement of outcome	Selective reporting of outcome
Arya [48]	India	2019	Low-Mid	Low	High (no date of intervention)	Low	Low	Low-Mid some pesticide suicides were classified as other poisoning suicides)	Low
Chowdhury [5]	Bangladesh	2018	Low-Mid	Low	Low	Low	Low	Low	Low-Mid (reported a decline in overall unnatural deaths, not suicide)
Kreitman [49]	England, Wales	1976	Low-Mid	Low	Low	Low	Low	Low	Low
Kreitman [50]	England, Wales	1984	Low-Mid	Low	Low	Low	Low	Low	Mid (limited reporting of outcome)
Gunnell [51]	England, Wales	2000	Low-Mid	Low	Low	Low	Low	Low	Low
Hassall [52]	Birmingham (England)	1972	Mid (limited pre-intervention data)	Mid (one city only)	Low	Low	Low	Low	Low
Lester [53]	Scotland	1992	Low-Mid (some trend analysis)	Low	Low	Low	Low	Low	Low
Curran [17]	Northern Ireland	1991	Mid (limited pre-intervention data)	Low	Low	Low	Low	Low	Low
Clarke [16]	Netherlands	1989	Low-Mid	Low	Low	Low	Low	Low	Low
Dervic [11]	Vienna (Austria)	2006	Low-Mid	Mid-High (only minors in one region)	Low	Low	Low	Low	Mid (reports changes over different periods, eg

© 2021 Lim JS et al. *JAMA Health Forum.*

Author	Location	Year of publica-tion	Risk of bias due to confounding	Selection bias of participants	Bias in classification of intervention	Bias due to deviations from intended interventions	Bias due to missing data	Bias in measurement of outcome	Selective reporting of outcome
									1956-1965 for gas suicides, 1953-1962 for overall suicide)
Wiedenmann [14]	West Germany	1993	Mid-High (no pre-intervention analysis)	Low	Low	Low	Low	Low	High (repeated cross sectional)
Moens [13]	Belgium	1989	Mid-High (no pre-intervention analysis)	Low	Low	Low	Low	Low	Mid (before and after study, missing some trend analysis)
Lester [15]	Switzerland	1990	Low-Mid	Low	Low	Mid (gas detoxification still being phased in at end of study period)	Low	Low	Low
Lester (domestic gas) [12]	Japan	1989	Low-Mid	Low	High (intervention start date not specified)	Low	Low	Low	Low
Lester (domestic gas) [10]	United States of America	1990	Low-Mid	Low	High (intervention start date not specified)	Low	Low	Low	Low
Burvill [18]	Australia	1989	Low-Mid	Low	High (intervention start date not specified)	Low	Low	Mid (all gas suicides by any cause were combined before 1950, then separated by domestic/motor/ other)	Low
Amos [23]	England, Wales	2001	Low-Mid	Low	Low	Low	Low	Low	Low

© 2021 Lim JS et al. *JAMA Health Forum.*

Author	Location	Year of publica-tion	Risk of bias due to confounding	Selection bias of participants	Bias in classification of intervention	Bias due to deviations from intended interventions	Bias due to missing data	Bias in measurement of outcome	Selective reporting of outcome
Thomas [54]	England, Wales	2010	Low-Mid	Low	Low	Low	Low	High (variation of classification by coroners from 1861-2007)	Low
Kendell [55]	England, Wales, Scotland	1998	High (case numbers reported with no analysis)	Low	Low	Mid-High (intervention still ongoing, only 36% of cars had catalytic converters)	Low	Low	Mid (only reports non-domestic gas suicide)
Skilling [24]	Scotland	2008	Low-Mid	Low	Low	Low	Low	Low	Low-Mid (reports median suicide rates before and after intervention instead of time trend)
Hepp [22]	Switzerland	2010	Low-Mid	Low	Low	Low	Low	Low	Low
Lester (motor) [25]	Japan	1989	Low-Mid	Low	Low	Low	Low	Low	Low-Mid (only 6 years follow-up after intervention)
Lester (motor) [56]	United States of America	1989	Low-Mid	Low	High (unspecified intervention, recommendation for catalytic converter but not formal)	Low	Low	Low	Low
Mott [21]	United States of America	2002	Low-Mid	Low	Low	Low	Low	Low	Low

© 2021 Lim JS et al. *JAMA Health Forum.*

Author	Location	Year of publica-tion	Risk of bias due to confounding	Selection bias of participants	Bias in classification of intervention	Bias due to deviations from intended interventions	Bias due to missing data	Bias in measurement of outcome	Selective reporting of outcome
Hampson [57]	United States of America	2015	Low-Mid	Low	Low	Low	Low	Mid (data from 2 centres)	Low
Routley [26]	Australia	1998	Low-Mid	Low	Low	Low	Low	Low	Low
Janik [58]	Hradec Kralove (Czech republic)	2017	Low-Mid	Mid (single institute)	High (intervention start date not specified)	Low	Low	Mid (some missing data 1980-1989)	Mid (reports general carbon monoxide suicides)
Hawton [59]	England, Wales	2004	Low	Low	Low	Low	Low	Low-Mid (includes open verdict deaths)	Low
Hawton [30]	England, Wales	2013	Low	Low	Low	Low	Low	Low-Mid (includes open verdict deaths)	Low
Morgan [60]	England, Wales	2007	Low	Low	Low	Low	Low	Mid (uses mortality data, not clear if this is suicide data)	Low
Hawton [61]	England, Wales	2009	Low	Low	Low	Low	Low	Low-Mid (includes open verdict deaths)	Mid (only 2 years follow-up after intervention)
Hawton [31]	England, Wales	2012	Low	Low	Low	Low	Low	Mid (study only uses single drug poisoning)	Low
Sandilands [62]	Scotland	2008	Mid (limited trend analysis)	Low	Low	Mid (intervention still ongoing)	Low	Mid (uses mortality data, not clear if this is suicide data)	Low
Delcher [63]	Florida (United States of America)	2017	Low-Mid	Mid (one city only)	Low	Mid (prescriptions slowed before intervention)	Low	Low	Low

© 2021 Lim JS et al. *JAMA Health Forum.*

Author	Location	Year of publica-tion	Risk of bias due to confounding	Selection bias of participants	Bias in classification of intervention	Bias due to deviations from intended interventions	Bias due to missing data	Bias in measurement of outcome	Selective reporting of outcome
Carlsten [27]	Sweden	1996	Low-Mid	Low	Low	Low	Low	Low	Low
Oliver [29]	Australia	1972	Low-Mid	Low	Low	Low	Low	Low	High (only reports general drug suicides)
Whitlock [64]	Brisbane (Australia)	1975	Low-Mid	Mid (one city only)	Low	Low	Low	Low	High (claims carbon monoxide suicide decreases but no data provided)
Thelander [65]	Sweden	2010	High (case numbers reported with no analysis)	Low	Low	Low	Low	Low	Low
Nordentoft [19]	Denmark	2006	Low-Mid	Low	Mid (overlap of interventions, no details of dextropropoxyphene restriction)	Low	Low	Low	Low-Mid (reports analgesic suicides for dextropropoxyphene restriction)
Nordentoft [20]	Denmark	2007	Low-Mid	Low	Mid (overlap of interventions, no details of dextropropoxyphene restriction)	Low	Low	Low	Low-Mid (reports analgesic suicides for dextropropoxyphene restriction)

© 2021 Lim JS et al. *JAMA Health Forum.*

eTable 2. Approximate or Estimated Number Of Annual Poison-Specific Suicides Reported One Year Before Each Intervention (or Closest Year Available). This represents the cohort who would benefit the most from means restriction of poisons.

Note: not enough information from Hradec Kralove (Czech republic) to be included in this table

LOCATION	POISON CLASS	YEAR	ANNUAL NUMBER OF POISON-SPECIFIC SUICIDES REPORTED BEFORE THE INTERVENTION
**USA**	Domestic gas	1950	1105
**SWITZERLAND**	Domestic gas	1955	177
**ENGLAND, WALES**	Domestic gas	1958	2499
**AUSTRALIA**	Domestic gas	1960	136
**FINLAND**	Pesticide	1960	75
**JAPAN**	Medicine (barbiturate)	1961	5180
**NETHERLANDS**	Domestic gas	1963	167
**SCOTLAND**	Domestic gas	1963	219
**WEST GERMANY**	Domestic gas	1963	1674
**NORTHERN IRELAND**	Domestic gas	1964	21
**VIENNA (AUSTRIA)**	Domestic gas	1965	9
**BELGIUM**	Domestic gas	1966	336
**AUSTRALIA**	Medicine (barbiturate)	1967	823
**JAPAN**	Domestic gas	1970	1156
**ENGLAND, WALES, SCOTLAND**	Pesticide	1972	10
**JAPAN**	Motor exhaust	1975	661
**USA**	Motor exhaust	1975	2101
**IRELAND**	Pesticide	1975	7
**DENMARK**	Domestic gas	1980	46
**TAIWAN**	Pesticide	1980	611
**GERMANY**	Pesticide	1984	499
**SRI LANKA**	Pesticide	1984	1855
**SWEDEN**	Medicine (barbiturate)	1985	96
**DENMARK**	Medicine (barbiturate)	1986	59
**SWITZERLAND**	Motor exhaust	1986	128
**AUSTRALIA**	Motor exhaust	1986	331
**DENMARK**	Medicine (dextropropoxyphene)	1987	101
**DENMARK**	Motor exhaust	1989	142
**HUNGARY**	Pesticide	1990	312
**ENGLAND, WALES**	Motor exhaust	1993	1304
**SCOTLAND**	Motor exhaust	1993	108
**ENGLAND, WALES**	Medicine (paracetamol)	1998	149
**BANGLADESH**	Pesticide	2000	7600
**INDIA**	Pesticide	2001	22000
**CRETE (GRECE)**	Pesticide	2003	8
**ENGLAND, WALES**	Medicine (dextropropoxyphene)	2005	189

© 2021 Lim JS et al. *JAMA Health Forum.*

LOCATION	POISON CLASS	YEAR	ANNUAL NUMBER OF POISON-SPECIFIC SUICIDES REPORTED BEFORE THE INTERVENTION
**SCOTLAND**	Medicine (dextropropoxyphene)	2005	37
**MARSEILLE (FRANCE)**	Pesticide	2007	2
**SRI LANKA**	Pesticide	2009	2373
**FLORIDA (USA)**	Medicine (propoxyphene)	2010	155
**INNER MONGOLIA (CHINA)**	Pesticide	2011	314
**SOUTH KOREA**	Pesticide	2011	2580
**SUM**			**57355**

© 2021 Lim JS et al. *JAMA Health Forum.*

eMethods 3. Excluded Papers With Reasons

Excluded During Full-Text Screening (from the Search Strategy):

Article	Reasons
Abdullat, E. M.; Hadidi, M. S.; Alhadidi, N.; Al-Nsour, T. S.; Hadidi, K. A. Agricultural and horticultural pesticides fatal poisoning; the Jordanian experience 1999-2002. J 2006;13(6-8):304-7	Suicide is NOT reported as a trend over time
Anonymous. UK withdrawal of co-proxamol delivers poisoning mortality reduction. Australian Journal of Pharmacy 2009;90(1074):78	Magazine/not a journal article
Bateman, D. N. Limiting paracetamol pack size: has it worked in the UK? Clin Toxicol (Phila) 2009;47(6):536-41	Literature review
Brown, J. H. Suicide in Britain. More attempts, fewer deaths, lessons for public policy. Arch Gen Psychiatry 1979;36(10):1119-24	Literature review
Cha, E. S.; Chang, S. S.; Choi, Y.; Lee, W. J. Trends in pesticide suicide in South Korea, 1983-2014. Epidemiology and Psychiatric Sciences 2020;29():	Duplicate
Chang, S. S.; Cheng, Q.; Lee, E. S.; Yip, P. S. Suicide by gassing in Hong Kong 2005-2013: Emerging trends and characteristics of suicide by helium inhalation. J Affect Disord 2016;192():162-6	No intervention
Clarke, M. J. Suicides by opium and its derivatives, in England and Wales, 1850–1950. Psychological Medicine 1985;15(2):237-242	No intervention
Dyvesether, S. M.; Nordentoft, M.; Forman, J. L.; Erlangsen, A. Joinpoint regression analysis of suicides in Denmark during 1980-2015. Danish Medical Journal 2018;65(4):	Suicide reported but NOT by poison
Etemadi-Aleagha, A.; Akhgari, M.; Iravani, F. S. Aluminum Phosphide Poisoning-Related Deaths in Tehran, Iran, 2006 to 2013. Medicine 2015;94(38):	Suicide is NOT reported as a trend over time
Flanagan, R. J. Fatal toxicity of drugs used in psychiatry. Human Psychopharmacology 2008;23(SUPPL. 1):43-51	Fatal poisoning, NOT suicidal intent
Fountain, J. S.; Reith, D. M.; Tomlin, A. M.; Smith, A. J.; Tilyard, M. W. Deaths by poisoning in New Zealand, 2008-2013. Clinical Toxicology 2019;57(11):1087-1094	Fatal poisoning, NOT suicidal intent
Gorman, D. R.; Bain, M.; Inglis, J. H. C.; Murphy, D.; Bateman, D. N. How has legislation restricting paracetamol pack size affected patterns of deprivation related inequalities in self-harm in Scotland? Public Health 2007;121(1):45-50	Fatal poisoning, NOT suicidal intent
Griffiths, C.; Flanagan, R. J. Fatal poisoning with antipsychotic drugs, England and Wales 1993-2002. J Psychopharmacol 2005;19(6):667-74	Fatal poisoning, NOT suicidal intent

© 2021 Lim JS et al. *JAMA Health Forum.*

Hampson, N. B. US Mortality Due to Carbon Monoxide Poisoning, 1999-2014 Accidental and Intentional Deaths. Annals of the American Thoracic Society 2016;13(10):1768-1774	Study period is well after the intervention (not suitable for before and after comparison)
Handley, S.; Patel, M. X.; Flanagan, R. J. Antipsychotic-related fatal poisoning, England and Wales, 1993-2013: impact of the withdrawal of thioridazine. Clin Toxicol (Phila) 2016;54(6):471-80	Fatal poisoning, NOT suicidal intent
Kaa, E.; Gregersen, M. Fatal poisonings in Jutland (Denmark) during the 1980s. Int J Legal Med 1992;105(3):133-8	Fatal poisoning, NOT suicidal intent
Kelly, S.; Bunting, J. Trends in suicide in England and Wales, 1982-96. Popul Trends 1998;(92):29-41	Magazine/not a journal article
Kim, H.; Kwon, S. W.; Ahn, Y. M.; Jeon, H. J.; Park, S.; Hong, J. P. Implementation and outcomes of suicide-prevention strategies by restricting access to lethal suicide methods in Korea. J Public Health Policy 2019;40(1):91-102	Literature review
McLaughlin, C.; Whittington, D. Suicide in Northern Ireland: a comparison of two quinquennia (1982-1986 and 1987-1991). J Psychiatr Ment Health Nurs 1996;3(1):13-20	Study period is well after the intervention (not suitable for before and after comparison)
McLoone, P.; Crombie, I. K. Trends in suicide in scotland 1974-84: An increasing problem. British Medical Journal (Clinical research ed.) 1987;295(6599):629-631	Study period is well after the intervention (not suitable for before and after comparison)
Morgan, O.; Griffiths, C.; Majeed, A. Impact of paracetamol pack size restrictions on poisoning from paracetamol in England and Wales: an observational study. Journal of Public Health 2005;27(1):19-24	Fatal poisoning, NOT suicidal intent
Morrell, S.; Page, A. N.; Taylor, R. J. The decline in Australian young male suicide. Social Science & Medicine 2007;64(3):747-754	No intervention
Nielsen, A. S.; Nielsen, B. Pattern of choice in preparation of attempted suicide by poisoning--with particular reference to changes in the pattern of prescriptions. [Danish] Ugeskrift for laeger 1992;154(28):1972-1976	Attempted suicide, NOT suicide
Page, A.; Liu, S.; Gunnell, D.; Astell-Burt, T.; Feng, X.; Wang, L.; Zhou, M. Suicide by pesticide poisoning remains a priority for suicide prevention in China: Analysis of national mortality trends 2006-2013. J Affect Disord 2017;208():418-423	No intervention
Sircar, K.; Clower, J.; Shin, M. K.; Bailey, C.; King, M.; Yip, F. Carbon monoxide poisoning deaths in the United States, 1999 to 2012. Am J Emerg Med 2015;33(9):1140-5	Study period is well after the intervention (not suitable for before and after comparison
Soltaninejad, K.; Nelson, L. S.; Bahreini, S. A.; Shadnia, S. Fatal aluminum phosphide poisoning in Tehran-Iran from 2007 to 2010. Indian J Med Sci 2012;66(3-4):66-70	Suicide is NOT reported as a trend over time

© 2021 Lim JS et al. *JAMA Health Forum.*

Studdert, D. M.; Gurrin, L. C.; Jatkar, U.; Pirkis, J. Relationship between vehicle emissions laws and incidence of suicide by motor vehicle exhaust gas in Australia, 2001-06: an ecological analysis. PLoS Med 2010;7(1):e1000210	Study period is well after the intervention (not suitable for before and after comparison)
Varnik, A.; Kolves, K.; Vali, M.; Tooding, L. M.; Wasserman, D. Do alcohol restrictions reduce suicide mortality? Addiction 2007;102(2):251-6	Suicide reported but NOT by poison
Wasserman, D.; Varnik, A.; Eklund, G. Male suicides and alcohol consumption in the former USSR. Acta Psychiatr Scand 1994;89(5):306-13	Suicide reported but NOT by poison

Other articles of interest which did not meet the selection criteria:

Article	Reasons
Bowles JR. Suicide in Western Samoa—an example of a suicide prevention program in a developing country. In: Diekstra R F Wea, ed. Preventive strategies on suicide. Leiden: Brill; 1995. 173–206	No intervention (availability of pesticide dropped for economic reasons)
Chaparro-Narvaez P, Castaneda-Orjuela C. Mortality due to pesticide poisoning in Colombia, 1998-2011. [Spanish] Biomedica 2015; 35: 90–102.	Fatal poisoning, NOT suicidal intent
Frost J, Poulsen E. Poisoning due to parathion and other organophosphorus insecticides in Denmark. Dan Med Bull 1964; 11: 169–77.	Fatal poisoning, NOT suicidal intent
Gunnell D, Coope C, Fearn V, Wells C, Chang SS, Hawton K, et al. Suicide by gases in England and Wales 2001-2011: evidence of the emergence of new methods of suicide. J Affect Disord. 2015;170:190-5	Study period is well after the intervention (not suitable for before and after comparison)
Ito T, Nakamura Y. Deaths from Pesticide Poisoning in Japan, 1968-2005: Data from Vital Statistics. Journal of Rural Medicine. 2008;3(1):5-9.	Fatal poisoning, NOT suicidal intent
Pearson M, Metcalfe C, Jayamanne S, Gunnell D, Weerasinghe M, Pieris R, et al. Effectiveness of household lockable pesticide storage to reduce pesticide self-poisoning in rural Asia: a community-based, cluster-randomised controlled trial. The Lancet. 2017;390(10105):1863-72.	Community based trial, not national intervention

© 2021 Lim JS et al. *JAMA Health Forum.*

eReferences

1. LaMorte WW. Comparing Frequencies: Rate Ratios. 2018; https://sphweb.bumc.bu.edu/otlt/MPH-Modules/PH717-QuantCore/PH717_ComparingFrequencies/PH717_ComparingFrequencies9.html. Accessed 14012021.
2. Moher D, Liberati A, Tetzlaff J, Altman DG. Preferred reporting items for systematic reviews and meta-analyses: the PRISMA statement. *BMJ.* 2009;339:b2535.
3. Knipe DW, Chang SS, Dawson A, et al. Suicide prevention through means restriction: Impact of the 2008-2011 pesticide restrictions on suicide in Sri Lanka. *PLoS ONE.* 2017;12(3).
4. Kastanaki AE, Kraniotis CF, Kranioti EF, Nathena D, Theodorakis PN, Michalodimitrakis M. Suicide by pesticide poisoning: Findings from the island of Crete, Greece. *Crisis: The Journal of Crisis Intervention and Suicide Prevention.* 2010;31(6):328-334.
5. Chowdhury FR, Dewan G, Verma VR, et al. Bans of WHO Class I Pesticides in Bangladesh-suicide prevention without hampering agricultural output. *International Journal of Epidemiology.* 2018;47(1):175-184.
6. Qin P, Du M, Wang S, et al. The waterfall pattern of suicide mortality in Inner Mongolia for 2008-2015. *Journal of Affective Disorders.* 2019;256:331-336.
7. Ohberg A, Lonnqvist J, Sarna S, Vuori E, Penttila A. Trends and availability of suicide methods in Finland. Proposals for restrictive measures. *Br J Psychiatry.* 1995;166(1):35-43.
8. Moebus S, Bödeker W. Mortality of intentional and unintentional pesticide poisonings in Germany from 1980 to 2010. *Journal of public health policy.* 2015;36(2):170-180.
9. Cha ES, Chang SS, Gunnell D, Eddleston M, Khang YH, Lee WJ. Impact of paraquat regulation on suicide in South Korea. *International Journal of Epidemiology.* 2016;45(2):470-479.
10. Lester D. The effects of detoxification of domestic gas on suicide in the United States. *Am J Public Health.* 1990;80(1):80-81.
11. Dervic K, Friedrich E, Prosquill D, et al. Suicide among Viennese minors, 1946-2002. *Wiener Klinische Wochenschrift.* 2006;118(5-6):152-159.
12. Lester D, Abe K. The effect of restricting access to lethal methods for suicide: a study of suicide by domestic gas in Japan. *Acta Psychiatrica Scandinavica.* 1989;80(2):180-182.
13. Moens GFG, Loysch MJM, Honggokoesoemo S, van de Voorde H. Recent trends in methods of suicide. *Acta Psychiatrica Scandinavica.* 1989;79(3):207-215.
14. Wiedenmann A, Weyerer S. The impact of availability, attraction and lethality of suicide methods on suicide rates in Germany. *Acta Psychiatrica Scandinavica.* 1993;88(5):364-368.
15. Lester D. The effect of the detoxification of domestic gas in Switzerland on the suicide rate. *Acta Psychiatrica Scandinavica.* 1990;82(5):383-384.
16. Clarke RV, Mayhew PAT. Crime as opportunity: a note on domestic gas suicide in Britain and the Netherlands. *The British Journal of Criminology.* 1989;29(1):35-46.
17. Curran PS, Lester D. Trends in the methods used for suicide in Northern Ireland. *Ulster Med J.* 1991;60(1):58-62.
18. Burvill PW. The changing pattern of suicide by gassing in Australia, 1910-1987: the role of natural gas and motor vehicles. *Acta Psychiatr Scand.* 1990;81(2):178-184.
19. Nordentoft M, Qin P, Helweg-Larsen K, Juel K. Time-trends in method-specific suicide rates compared with the availability of specific compounds. The Danish experience. *Nord J Psychiatry.* 2006;60(2):97-106.
20. Nordentoft M, Qin P, Helweg-Larsen K, Juel K. Restrictions in means for suicide: an effective tool in preventing suicide: the Danish experience. *Suicide Life Threat Behav.* 2007;37(6):688-697.
21. Mott JA, Wolfe MI, Alverson CJ, et al. National vehicle emissions policies and practices and declining US carbon monoxide-related mortality. *JAMA.* 2002;288(8):988-995.
22. Hepp U, Ring M, Frei A, Rossler W, Schnyder U, Ajdacic-Gross V. Suicide trends diverge by method: Swiss suicide rates 1969-2005. *Eur Psychiatry.* 2010;25(3):129-135.
23. Amos T, Appleby L, Kiernan K. Changes in rates of suicide by car exhaust asphyxiation in England and Wales. *Psychological Medicine.* 2001;31(5):935-939.

© 2021 Lim JS et al. *JAMA Health Forum.*

24. Skilling GD, Sclare PD, Watt SJ, Fielding S. The effect of catalytic converter legislation on suicide rates in Grampian and Scotland 1980-2003. *Scottish medical journal.* 2008;53(4):3-6.
25. Lester S, Abe K. Car availability, exhaust toxicity, and suicide. *Annals of Clinical Psychiatry.* 1989;1(4):247-250.
26. Routley VH, Ozanne-Smith J. The impact of catalytic converters on motor vehicle exhaust gas suicides. *Medical Journal of Australia.* 1998;168(2):65-67.
27. Carlsten A, Allebeck P, Brandt L. Are suicide rates in Sweden associated with changes in the prescribing of medicines? *Acta Psychiatrica Scandinavica.* 1996;94(2):94-100.
28. Lester D, Abe K. The effect of Controls on Sedatives and Hypnotics on their use for Suicide. *Journal of Toxicology: Clinical Toxicology.* 1989;27(4-5):299-303.
29. Oliver RG, Hetzel BS. Rise and fall of suicide rates in Australia: relation to sedative availability. *The Medical journal of Australia.* 1972;2(17):919-923.
30. Hawton K, Bergen H, Simkin S, et al. Long term effect of reduced pack sizes of paracetamol on poisoning deaths and liver transplant activity in England and Wales: interrupted time series analyses. *BMJ.* 2013;346:f403.
31. Hawton K, Bergen H, Simkin S, Wells C, Kapur N, Gunnell D. Six-year follow-up of impact of co-proxamol withdrawal in England and Wales on prescribing and deaths: time-series study. *PLoS Med.* 2012;9(5):e1001213.
32. Sterne JAC, Hernán MA, Reeves BC, et al. ROBINS-I: a tool for assessing risk of bias in non-randomised studies of interventions. *BMJ.* 2016;355:i4919.
33. Onyon LJ, Volans GN. The Epidemiology and Prevention of Paraquat Poisoning. *Human & Experimental Toxicology.* 1987;6(1):19-29.
34. Fitzgerald GR, Barniville G, Flanagan M, Silke B, Carmody M, O'Dwyer WF. The changing pattern of paraquat poisoning: an epidemiologic study. *Irish medical journal.* 1978;71(4):103-108.
35. Berecz R, Caceres M, Szlivka A, et al. Reduced completed suicide rate in Hungary from 1990 to 2001: Relation to suicide methods. *Journal of Affective Disorders.* 2005;88(2):235-238.
36. Kervegant M, Merigot L, Glaizal M, Schmitt C, Tichadou L, de Haro L. Paraquat poisonings in France during the European ban: experience of the Poison Control Center in Marseille. *J Med Toxicol.* 2013;9(2):144-147.
37. Cha ES, Chang SS, Choi Y, Lee WJ. Trends in pesticide suicide in South Korea, 1983-2014. *Epidemiology and psychiatric sciences.* 2019;29:e25.
38. Kim J, Shin SD, Jeong S, Suh GJ, Kwak YH. Effect of prohibiting the use of Paraquat on pesticide-associated mortality. *BMC Public Health.* 2017;17(1):858.
39. Han DG, Kang SG, Cho SJ, Cho SE, Na KS. Suicide Methods According to Age and Sex: An Analysis of Data of 239,565 Suicide Victims in the Republic of Korea From 1991 to 2015. *J Nerv Ment Dis.* 2018;206(10):770-775.
40. Myung W, Lee GH, Won HH, et al. Paraquat prohibition and change in the suicide rate and methods in South Korea. *PLoS ONE.* 2015;10(6):e0128980.
41. Lee JW, Hwang IW, Kim JW, et al. Common Pesticides Used in Suicide Attempts Following the 2012 Paraquat Ban in Korea. *Journal of Korean medical science.* 2015;30(10):1517-1521.
42. Chang SS, Lu TH, Eddleston M, et al. Factors associated with the decline in suicide by pesticide poisoning in Taiwan: a time trend analysis, 1987-2010. *Clin Toxicol (Phila).* 2012;50(6):471-480.
43. Lin JJ, Lu TH. Trends in solids/liquids poisoning suicide rates in Taiwan: a test of the substitution hypothesis. *BMC Public Health.* 2011;11:712.
44. Chen YY, Kwok CL, Yip PS, Wu KC. A test of the substitution hypothesis: an analysis of urban and rural trends in solid/liquid poisoning suicides in Taiwan. *Soc Sci Med.* 2013;96:45-51.
45. Gunnell D, Fernando R, Hewagama M, Priyangika WD, Konradsen F, Eddleston M. The impact of pesticide regulations on suicide in Sri Lanka. *International Journal of Epidemiology.* 2007;36(6):1235-1242.
46. de Silva VA, Senanayake SM, Dias P, Hanwella R. From pesticides to medicinal drugs: time series analyses of methods of self-harm in Sri Lanka. *Bulletin of the World Health Organization.* 2012;90(1):40-46.
47. Knipe DW, Metcalfe C, Fernando R, et al. Suicide in Sri Lanka 1975-2012: age, period and cohort analysis of police and hospital data. *BMC Public Health.* 2014;14:839.
48. Arya V, Page A, Gunnell D, et al. Suicide by hanging is a priority for suicide prevention: method specific suicide in India (2001-2014). *Journal of Affective Disorders.* 2019;257:1-9.

© 2021 Lim JS et al. *JAMA Health Forum.*

49. Kreitman N. The coal gas story. United Kingdom suicide rates, 1960-71. *Br J Prev Soc Med.* 1976;30(2):86-93.
50. Kreitman N, Platt S. Suicide, unemployment, and domestic gas detoxification in Britain. *Journal of Epidemiology and Community Health.* 1984;38(1):1-6.
51. Gunnell D, Middleton N, Frankel S. Method availability and the prevention of suicide - a re-analysis of secular trends in England and Wales 1950-1975. *Social Psychiatry and Psychiatric Epidemiology.* 2000;35(10):437-443.
52. Hassall C, Trethowan WH. Suicide in Birmingham. *Br Med J.* 1972;1(5802):717-718.
53. Lester D, Hodgson J. The effects of the detoxification of domestic gas on the suicide rate in Scotland. *The European Journal of Psychiatry.* 1992;6(3):171-174.
54. Thomas K, Gunnell D. Suicide in England and Wales 1861-2007: a time-trends analysis. *International Journal of Epidemiology.* 2010;39(6):1464-1475.
55. Kendell RE. Catalytic converters and prevention of suicides. *Lancet.* 1998;352(9139):1525.
56. Lester D. Changing rates of suicide by car exhaust in men and women in the United States after car exhaust was detoxified. *Crisis: The Journal of Crisis Intervention and Suicide Prevention.* 1989;10(2):164-168.
57. Hampson NB, Holm JR. Suicidal carbon monoxide poisoning has decreased with controls on automobile emissions. *Undersea and Hyperbaric Medicine.* 2015;42(2):159-164.
58. Janik M, Ublova M, Kucerova S, Hejna P. Carbon monoxide-related fatalities: A 60-year single institution experience. *J Forensic Leg Med.* 2017;48:23-29.
59. Hawton K, Simkin S, Deeks J, et al. UK legislation on analgesic packs: before and after study of long term effect on poisonings. *BMJ.* 2004;329(7474):1076-1079.
60. Morgan OW, Griffiths C, Majeed A. Interrupted time-series analysis of regulations to reduce paracetamol (acetaminophen) poisoning. *PLoS Med.* 2007;4(4):e105.
61. Hawton K, Bergen H, Simkin S, et al. Effect of withdrawal of co-proxamol on prescribing and deaths from drug poisoning in England and Wales: time series analysis. *BMJ.* 2009;338:b2270.
62. Sandilands EA, Bateman DN. Co-proxamol withdrawal has reduced suicide from drugs in Scotland. *Br J Clin Pharmacol.* 2008;66(2):290-293.
63. Delcher C, Chen G, Wang Y, Slavova S, Goldberger BA. Fatal poisonings involving propoxyphene before and after voluntary withdrawal from the United States' market: An analysis from the state of Florida. *Forensic Sci Int.* 2017;280:228-232.
64. Whitlock FA. Suicide in Brisbane, 1956 to 1973: the drug death epidemic. *Medical Journal of Australia.* 1975;1(24):737-743.
65. Thelander G, Jonsson AK, Personne M, Forsberg GS, Lundqvist KM, Ahlner J. Caffeine fatalities--do sales restrictions prevent intentional intoxications? *Clin Toxicol (Phila).* 2010;48(4):354-358.

© 2021 Lim JS et al. *JAMA Health Forum.*
